# DNA-binding determinants promoting NHEJ by human Polµ

**DOI:** 10.1093/nar/gks896

**Published:** 2012-10-02

**Authors:** Maria Jose Martin, Raquel Juarez, Luis Blanco

**Affiliations:** Department of Genome Dynamics and Function, Centro de Biologia Molecular Severo Ochoa (CSIC-UAM), 28049 Madrid, Spain

## Abstract

Non-homologous end-joining (NHEJ), the preferred pathway to repair double-strand breaks (DSBs) in higher eukaryotes, relies on a collection of molecular tools to process the broken ends, including specific DNA polymerases. Among them, Polµ is unique as it can catalyze DNA synthesis upon connection of two non-complementary ends. Here, we demonstrate that this capacity is intrinsic to Polµ, not conferred by other NHEJ factors. To understand the molecular determinants of its specific function in NHEJ, the interaction of human Polµ with DNA has been directly visualized by electromobility shift assay and footprinting assays. Stable interaction with a DNA gap requires the presence of a recessive 5′-P, thus orienting the catalytic domain for primer and nucleotide binding. Accordingly, recognition of the 5′-P is crucial to align the two DNA substrates of the NHEJ reaction. Site-directed mutagenesis demonstrates the relevance of three specific residues (Lys^249^, Arg^253^ and Arg^416^) in stabilizing the primer strand during end synapsis, allowing a range of microhomology-induced distortions beneficial for NHEJ. Moreover, our results suggest that the Polµ BRCT domain, thought to be exclusively involved in interaction with NHEJ core factors, has a direct role in binding the DNA region neighbor to the 5′-P, thus boosting Polµ-mediated NHEJ reactions.

## INTRODUCTION

Non-homologous end-joining (NHEJ) is the main mechanism to repair DSBs in higher eukaryotes, as it is operative throughout the cell cycle, unlike the second mechanism that serves to repair this type of damage, homologous recombination (HR), which is inhibited during the phases G_0_, G_1_ and S (1). Analysis of the sequences repaired by NHEJ at the break points suggests that some of these events involve the alignment of the ends through microhomologies (complementary sequences from 1 to 4 nt) near the site of rupture (2,3). When there is no direct microhomology, the repair system must generate it by allowing resection, distortions, flaps or gaps that may arise in the attempt to align the ends of the DSB [reviewed in (4)], thus implying the processing action of specific nucleases and/or DNA polymerases (5–7). The X family DNA polymerases Polµ (5,8–12) and Polλ (11,13–16) have been involved in the NHEJ process. An extreme NHEJ reaction would involve ends with minimal 3′-protrusions that are not compatible. Polµ is unique as it can bridge these ends with no further processing, catalyzing the necessary nucleotide insertions to close the gaps for further ligation and repair with a minimal loss of the original sequences. Moreover, Polµ can perform template-independent polymerization to create the necessary complementary sequences (11,17).

Crystallization studies of Polβ, the paradigm of the X family, showed that this polymerase is well suited for filling short gaps during DNA repair because it simultaneously binds not only the 5′-end through a specific pocket interacting with the 5′-phosphate group but also the 3′-end of short gaps through the fingers and palm subdomains. By comparing the corresponding crystal structures, it appears that these general features of the Polβ-like core are shared by the rest of the members of the X family, Polµ, Polλ and TdT (PDB IDs: 2IHM, 1XSN and 1KDH). As a complement to the 3D structural work, studying the DNA-binding properties of a DNA polymerase in solution is of central importance in order to elucidate its role in different cellular processes. Thus, both quantitative and qualitative analysis of binding to different DNA polymerization substrates can reflect intrinsic properties, such as processivity versus distributivity and intrinsic preferences for DNA repair versus DNA replication substrates. In this work, we have used *in vitro* assays that evidence the formation of stable DNA–protein complexes either in the absence or in the presence of nucleotide and metal ions necessary for catalysis. Moreover, these complexes were observed on different DNA substrates that mimic the structures generated in various DNA repair processes. Our results show that Polµ is specialized in binding small DNA gaps and also, and most importantly, substrates similar to those found in DSB repair reactions, and that this binding is completely dependent on the presence of a 5′-phosphate group and qualitatively enhanced by the formation of a ternary complex in solution. Furthermore, through the study of different point and deletion mutants, we uncovered a new DNA-binding function for the BRCT domain of Polµ and demonstrated the importance of several residues located at the primer-binding region, for both DNA-binding and polymerization activities. Our work dissects the ambidexterity of the active site of Polµ in terms of its ability to bind and orient two DNA ends without the help of any additional factors and the available solutions for a vast array of damaged and misaligned substrates, thus providing an in-depth insight of the intricacies of the NHEJ process.

## MATERIALS AND METHODS

### DNA and proteins

Synthetic DNA oligonucleotides were obtained from Isogen (Ijsselstein, Holland). Polyacrylamide gel electrophoresis (PAGE)-purified oligonucleotides were labeled at their 5′-ends with [γ-^32^P]ATP. The oligonucleotides used to generate the DNA substrates were the following: for 1-nt gapped substrates, Sp1C (5′-GATCACAGTGAGTAC-3′), T28 (5′-AGAAGTGTATCTCGTACTCACTGTGATC-3′) and D12 (5′-AGATACACTTCT-3′); for 2-nt gapped substrates, P15 (5′-TCTGTGCAGGTTCTT-3′), T32 (5′-TGAAGTCCCTCTCGACGAAGAACCTGCACAGA-3′) and D16 (5′-GTCGAGAGGGACTTCA-3′); as single-stranded DNA (ssDNA) for terminal transferase assays, polydA (5′-AAAAAAAAAAAAAAAAAAAAA-3′), to avoid the formation of secondary structures; for footprinting assays, oligonucleotides FP-P (5′-GGCCACGCAATGTTGACGTTTTTCGACAAGACCTCAGTAT-3′), FP-T (5′-GGCAGCTTGGATCTTGTCGAAAAACGTCAACATTCGCCTAGGCTTCGGCAATACTGAGGTCTTGTCGAAAAACGTCAACATTGCGTGGCC-3′) and FP-D (5′-GCCGAAGCCTAGGCGAATGTTGACGTTTTTCGACAAGATCCAAGCTGCC-3′). As a positive control of hybridization of the three footprint oligonucleotides, the gapped substrate conforms two consensus binding boxes for Spo0A [*Bacillus subtillis* sporulation factor; (18)], located at both the primer and downstream sides of the gap. For NHEJ assays, oligonucleotides CA (5′-CCCTCCCTCCCCA-3′), GT (5′-CCCTCCCTCCCGT-3′), G (5′-CCCTCCCTCCCG-3′) and C (5′-CCCTCCCTCCCC-3′) were used as primers, hybridized to oligonucleotide NHEJ-D (5′-GGGAGGGAGGG-3′). Also, oligonucleotides D3 (5′-CCCTCCCTCCGCGGC-3′), D3BB1 (5′-CCCTCCCTCCGCGGAC-3′), D3BB2 (5′-CCCTCCCTCCGCGAGC-3′), D3MM (5′-CCCTCCCTCCGCGAC-3′) and D3FLAP (5′-CCCTCCCTCCGCGGA-3′) were used as primers hybridized to D1 (5′-CGGAGGGAGGG-3′), while oligonucleotides D4 (5′-CGCGCACTCACGTCCCCGCC-3′), D4BB1 (5′-CGCGCACTCACGTCCCCGCAC-3′), D4BB2 (5′-CGCGCACTCACGTCCCCGACC-3′), D4MM (5′-CGCGCACTCACGTCCCCGAC-3′), D4FLAP (5′-CGCGCACTCACGTCCCCGCA-3′), D4AC (5′-CGCGCACTCACGTCCCCAGCC-3′), D4CC (5′-CGCGCACTCACGTCCCCCGCC-3′), D4GC (5′-CGCGCACTCACGTCCCCGGCC-3′), D4CT (5′-CGCGCACTCACGTCCCCTGCC–3′), D4CA (5′-CGCGCACTCACGTCCCACGCC-3′), D4CG (5′-CGCGCACTCACGTCCCGCGCC-3′) and D4CT (5′-CGCGCACTCACGTCCCTCGCC-3′) were hybridized to D2 (5′-GGGACGTGAGTGCGCG-3′). Oligonucleotides D12, D16, FP-D, NHEJ-D, D1 and D2 contain a 5′-P group when indicated. Ultrapure dNTPs, ddNTPs, [α-^32^P] dNTPs (3000 Ci/mmol) and [γ-^32^P]ATP (3000 Ci/mmol) were purchased from GE Healthcare (USA). T4 polynucleotide kinase was obtained from New England Biolabs (Beverly, MA, USA). *Pfu* DNA polymerase and DNAse I were purchased from Promega Corporation (Madison, WI, USA). Purified human Polλ was obtained as described (19).

### Construction and purification of human Polµ mutant proteins

Site-directed mutagenesis by single PCR with oligonucleotides containing the desired mutation was performed on the Polµ-overexpressing plasmid pRSETA-hPolµ (12). The oligonucleotides used were the following: K249AF (5′-GGGGTCGGTGTGGCGACTGCTGACCGG-3′), K249AR (5′-CCGGTCAGCAGTCGCCACACCGACCCC-3′), R253AF (5′-AAGACTGCTGACGCGTGGTACCGGGAA-3′), R253AR (5′-TTCCCGGTACCACGCGTCAGCAGTCTT-3′), R416AF (5′-TGGAAGGCCGTGGCAGTGGACTTGGTA-3′) and R416AR (5′-TACCAAGTCCACTGCCACGGCCTTCCA-3′). DNA constructs were sequenced and transformed in *Escherichia coli* BL21(DE3)pLysS. Wild-type and mutant Polµ variants were overexpressed and purified in an Äkta Purifier FPLC system (GE Healthcare) with the following protocol: the cleared bacterial lysate was loaded on a HiTrap heparin column and the selected eluted fractions were loaded on a HiTrap SP sepharose column to eliminate contaminant nucleases. The Polµ-ΔBRCT deletion mutant was obtained by amplification of the fragment corresponding to the Polβ-like core from the pZero-Polµ plasmid (20) using oligonucleotides h2(s)βNdeI (5′-GCCCAGCACATATGCCTGCCTATGCCT-3′) and h2(as)EcoRI (5′-CGGAATTCAGGCGTTTCTCTGCTC-3′), which introduce the restriction sites for NdeI and EcoRI, respectively. The PCR product was cloned on the bacterial expression vector pRSET-A (Invitrogen) to obtain the pRSETA-ΔBRCThPolµ construct. The recombinant protein of 40 KDa, containing amino acids 138–494 of human Polµ, was purified using the Äkta Purifier FPLC system with a slightly different protocol: the cleared bacterial lysate was loaded on S and Q sepharose columns in tandem, the flow-trough was then subjected to a second chromatography step on a heparin column, and the eluted fractions were loaded on a manually packed phosphocellulose column to eliminate contaminant nucleases. In all the cases, the eluted fractions containing highly purified protein were concentrated and stored at −80°C.

### Construction and purification of human Polβ

Polβ complementary DNA was obtained from the IMAGE consortium and subcloned in the pET16b bacterial overexpression plasmid. The final clone was transformed in *E. coli* BL21(DE3)pRil, cells were grown up to 0.6 OD (optical density). and Polβ expression was induced with 0.5 mM IPTG during 160 min. Cells were lysed with alumina and centrifuged for 15 min at 15 000 rpm to obtain a debris- and insoluble protein-free extract. DNA was precipitated with polyethylenimine and eliminated by centrifugation. Soluble proteins in the supernatant were precipitated with 65% ammonium sulfate. The pellet was resuspendend and subjected to chromatography in a 5 ml phosphocellulose column, followed by chromatography in a 5 ml heparin HiTrap column (Pharmacia Biotech). After a final concentration step in a mini phosphocellulose column (1 ml), the fractions containing highly purified Polβ were stored at −80°C.

### DNAseI footprinting assays

The indicated proteins at the designated concentrations were incubated with 30 nM labeled gapped substrate in a final volume of 20 µl, containing 50 mM Tris–HCl (pH 7.5), 1 mM DTT, 4% glycerol and 0.1 mg/ml of bovine serum albumin (BSA). After incubation for 10 min at 37°C, samples were treated with 0.03 units of commercial DNAseI for 2 min at 37°C. Reactions were stopped with a buffer containing 20 mM ethylenediaminetetraacetic acid (EDTA), and the DNA precipitated with 3 M sodium acetate and 100% EtOH, O/N at −80°C. The DNA pellets were washed with 70% EtOH and resuspended in loading buffer (10 mM EDTA, 95% [v/v] formamide, 0.03% [w/v] bromophenol blue, 0.03% [w/v] xylene cyanol), boiled and subjected to electrophoresis in 8 M urea-containing 8% polyacrylamide sequencing gels. Labeled DNA fragments were detected by autoradiography.

### Electromobility shift assays

Electromobility shift assays (EMSAs), used to analyze the interaction with gapped substrates and NHEJ intermediates, were performed in a final volume of 12.5 µl, containing 50 mM Tris–HCl (pH 7.5), 0.1 mg/ml of BSA, 1 mM DTT, 4% glycerol, 5 nM labeled DNA and different concentrations of the indicated proteins. After incubation for 10 min at 30°C, samples were mixed with 3 µl of 30% glycerol and resolved by native gel electrophoresis on a 4% polyacrylamide gel (80:1 [w/w] acrylamide/bisacrylamide). Gels were dried and labeled DNA fragments were detected by autoradiography. To calculate the binding affinity constants, the retarded and free DNA bands were quantified using ImageJ. Graphs representing log of complex/free DNA versus the log of enzyme concentration were used to calculate the KDs, defined as the intersection of the trendline with the *x*-axis.

### DNA polymerization assays

To analyze DNA-dependent and -independent polymerization, several DNA substrates, containing 5′P-labeled primers, were incubated with different proteins, at the concentration indicated in each case. The reaction mixture, in 20 µl, contained 50 mM Tris–HCl (pH 7.5), 1 mM DTT, 4% glycerol and 0.1 mg/ml BSA, in the presence of the indicated amounts of the DNA polymerization substrates and the indicated concentrations of dNTPs and activating metal ions. After incubation, reactions were stopped by adding gel loading buffer (95% [v/v] formamide, 10 mM EDTA, 0.1% [w/v] xylene cyanol and 0.1% [w/v] bromophenol blue) and analyzed by 8 M urea/20% PAGE and autoradiography. The data were fit to the Michaelis–Menten equation using non-linear regression. When indicated, ddNTPs were used instead of dNTPs to limit incorporation to a single nucleotide on the 3′-end of the labeled oligonucleotide.

### NHEJ assays

NHEJ polymerization assays were carried out essentially as described above for polymerization assays, but using independent DNA primer and template molecules. When indicated, variable concentrations of ddNTPs were used instead of dNTPs/NTPs to limit incorporation to a single nucleotide on the 3′-end of the labeled oligonucleotide.

### Amino acid sequence comparisons and 3D modeling

Multiple alignments of different DNA polymerases from the X family were done using the program MULTALIN (http://prodes.toulouse.inra.fr/multalin/). 3D coordinates for the structures used here were obtained from the Protein Data Bank (http://www.rcsb.org/pdb/). The different conformations of the studied residues in Polµ were analyzed by using the MacPymol software (http://delsci.com/macpymol/).

## RESULTS

### DNA-binding properties of human family X polymerases

**Figure 1.**
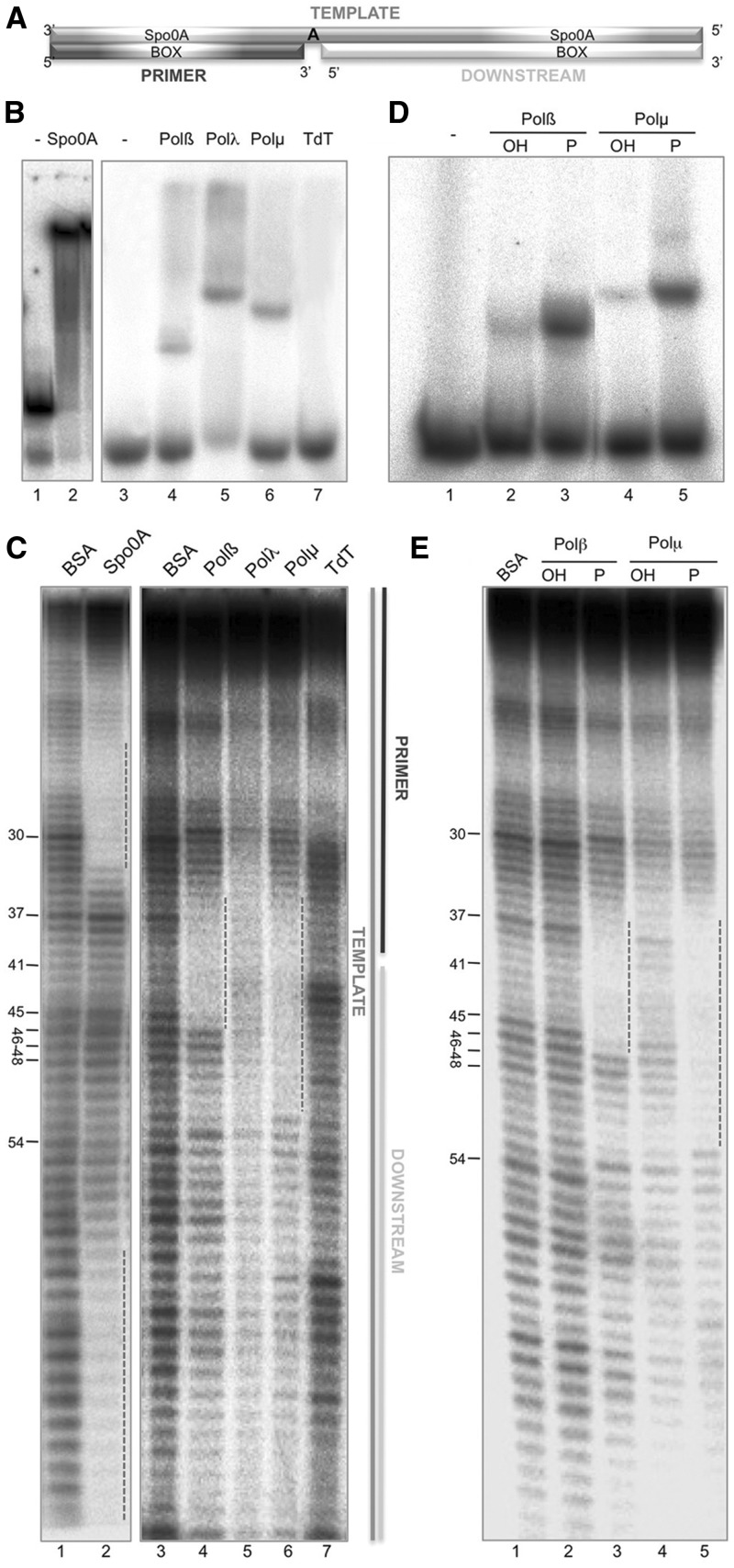
DNA-binding properties of human family X polymerases: importance of the 5′-P group for DNA binding. (**A**) Scheme of the substrates used for the footprinting assays. To produce this substrate the oligonucleotides FP-T (template), FP-P (primer) and FP-D (downstream) were hybridized. (**B**) DNA-binding affinity of the indicated proteins (Polβ and Polµ at 100 nM, Polλ at 500 nM) was assayed as described in ‘Materials and Methods’ section, using the footprinting substrate radioactively labeled at the 5′-end of the template strand. Gel was dried and the labeled fragments detected by autoradiography. (**C**) Footprinting assay of the control protein Spo0A (1 µg) or each of the members of the X family (Polβ, 1.5 µg; Polµ, 1.5 µg; Polλ, 10 µg, TdT, 10 µg) was conducted as described in ‘Materials and Methods’ section, in the presence of 100 µM dTTP and 2.5 mM MgCl_2_. Ten micrograms of BSA were added to the control lane. (**D**) EMSA of Polβ and Polµ (100 nM) using a gapped DNA substrate formed by the oligonucleotides Sp1C (labeled at the 5′-end), T28 and D12, the latter either having (P) of lacking (OH) a 5′-P group. Gel was dried and the labeled fragments detected by autoradiography. E) Footprinting assay of Polβ (1.5 µg) or Polµ (1.5 µg) with a gapped substrate that either contains (P) or lacks (OH) a 5′-P group in the downstream strand, in the presence of 100 µM dTTP and 2.5 mM MgCl_2_. Ten micrograms of BSA was added to the control lane. Gel was dried and the labeled fragments detected by autoradiography.

To achieve a better understanding of the DNA-binding capacities of the X family polymerases, we took advantage of the well-established EMSAs and also developed a footprinting assay that would allows us to further study the qualitative binding differences caused by the addition of nucleotide substrates or by the lack of certain residues or domains in the protein. The DNA substrates that are most commonly used for the DNAseI footprint technique are double-stranded, blunt-ended DNA fragments. However, these are not valid substrates for DNA polymerases, as they normally require ssDNA portions, recognized as the template strand. DNA polymerases from the X family are even more exquisite for binding, being short DNA gaps their preferred substrate. Thus, we decided to use a 1-nt gap containing substrate, produced by hybridization of three oligonucleotides: template (FP-T, 90-mer; radioactively labeled at its 5′-end), primer (FP-P, 40-mer) and downstream (FP-D, 49-mer), as shown in Figure 1A. Spo0A binding to its specific boxes (Figure 1A) was used as a positive control for hybridization of the tripartite substrate both in EMSA and footprint (Figure 1B, lane 2, and C, lane 2/dotted lines). The DNA-binding affinity of the four members of the human family X to this 1-nt gapped substrate was initially tested by EMSA. Both Polβ (100 nM; Figure 1B, lane 4) and Polµ (100 nM; Figure 1B, lane 6) formed stable DNA–protein complexes, while to obtain a similarly stable complex with Polλ the amount of polymerase needed was 5-fold higher (500 nM; Figure 1B, lane 5). In agreement with these results, both Polβ and Polµ produced a clear footprint on this DNA substrate (Figure 1C, lanes 4 and 6), being the protection caused by 5-fold higher amounts of Polλ visibly weaker (Figure 1C, lane 5). Our results indicate that both Polβ and Polµ show a strengthened binding to the DNA substrate than that of Polλ, as assessed both by EMSA and footprinting assays. The protections (indicated by a dotted line) are centered around the templating base, at position 41. As expected, TdT did not show a stable enzyme–DNA complex on this substrate, neither by EMSA (Figure 1B, lane 7) nor by footprinting (Figure 1C, lane 7), since this polymerase does not bind template-containing substrates. When comparing the footprint of Polβ with the crystal structures available of this polymerase bound to a gapped DNA substrate, we found a complete correspondence between the number of nucleotides (10 nt) protected in our assays [from position 36 to 45, both included: 5-nt upstream the gap (primer side), the templating base, and 4-nt downstream the gap] and the number of base pairs contacted by the polymerase in the 3D structure (Supplementary Figure 1A). In the case of Polµ, a similar protection was observed from position 36 to 45 (in agreement with the crystal structure of the Polµ core bound to a gap; Supplementary Figure 1B), but the footprint was further extended by ∼7 nt (up to position 52) toward the downstream part of the substrate. This extra protection is interpreted to be due to the presence of a BRCT domain in Polµ (20), absent in Polβ. Such a direct role of BRCT domains in DNA binding has been early proposed (21,22), but direct evidence of this for BRCT-containing DNA polymerases as Polλ, yeast Pol4, Polµ and TdT, and the benefit for NHEJ, had not been reported yet, as will be further discussed below.

### Importance of the 5′-P group for DNA binding

Previous studies with Polβ revealed that its ability to perform processive polymerization on a gapped DNA substrate of several nucleotides depends on the presence of a 5′-phosphate group flanking the gap (23). This 5′-P does have a positive influence on the catalytic efficiency of Polβ, as well as on the level of fidelity during synthesis, since both parameters are significantly increased when the 5′-P is present (24). These effects are promoted in part by an increase in the overall binding to DNA, mediated by the 8-kDa subdomain. Such an interaction, absent in replicative polymerases, is very specific as the 5′-P hallmarks the end point of polymerization. Polβ and Polµ have a conserved 8-kDa domain, and Polµ polymerization on a gapped substrate is also improved by the presence of the 5′-P (25). In fact, our results indicate that the catalytic efficiency of the reaction is increased due to a 5-fold reduction of the *K*m_app_, while the *K*_cat_ of the reaction remains unchanged (Supplementary Table S1). The quantitative data obtained are congruent with a major effect of the 5′-P as a DNA-binding determinant that improves the formation/stability of the enzyme–DNA binary complex. Consequently, templated selection of the incoming nucleotide is significantly improved. Despite this similar effect of the 5′-P group, these two polymerases show important differences regarding the number and electrostatic potential of the residues of the 8 kDa directly acting as 5′-P ligands (Supplementary Figure S1A and B, lower panels) that could be related to their intrinsic DNA-binding strength, or being a requisite for the intrinsic dRP lyase activity present in Polβ (26), but absent in Polµ. Therefore, it was important to determine the extent by which 5′-P binding directly contributes to Polµ DNA binding.

We compared the capacity of either Polβ or Polµ to bind gapped substrates which, when indicated, may contain or lack a 5′-P group in the downstream strand, thus flanking the gap. EMSA showed a stronger binding affinity of both Polβ and Polµ for the 5′-P-containing substrate (Figure 1D; compare lanes 2 and 3 for Polβ, and lanes 4 and 5 for Polµ). The binding affinity of Polµ to a gapped substrate is significantly improved by the presence of a 5′-P group, with a calculated KD value varying from 0.5 µM (5′-OH) to 0.1 µM (5′-P). Footprinting assays corroborated this result (Figure 1E; compare lanes 2 and 3 for Polβ, and lanes 4 and 5 for Polµ). In fact, footprints of Polβ and Polµ are hardly visible on the gapped substrate lacking the 5′-P. Thus, both polymerases, regardless their different number of amino acids interacting with the 5′-P, display a similar binding preference for gap-containing DNA substrates bearing a 5′-P, frequent intermediates in several DNA repair pathways.

### Ternary complex formation in solution

Analysis of the 3D information available shows structural differences in Polβ when forming binary (E:DNA; PDB ID: 1BPX) versus ternary (E:DNA:dNTP; PDB ID: 1BPY) complexes. To confirm that these differences exist in solution between the binary and ternary complexes of Polβ and Polµ, we decided to use our footprinting assay on a 1-nt gapped DNA either in the absence (binary) or in the presence (ternary) of deoxynucleotide (complementary or non-complementary to the templating base).

**Figure 2.**
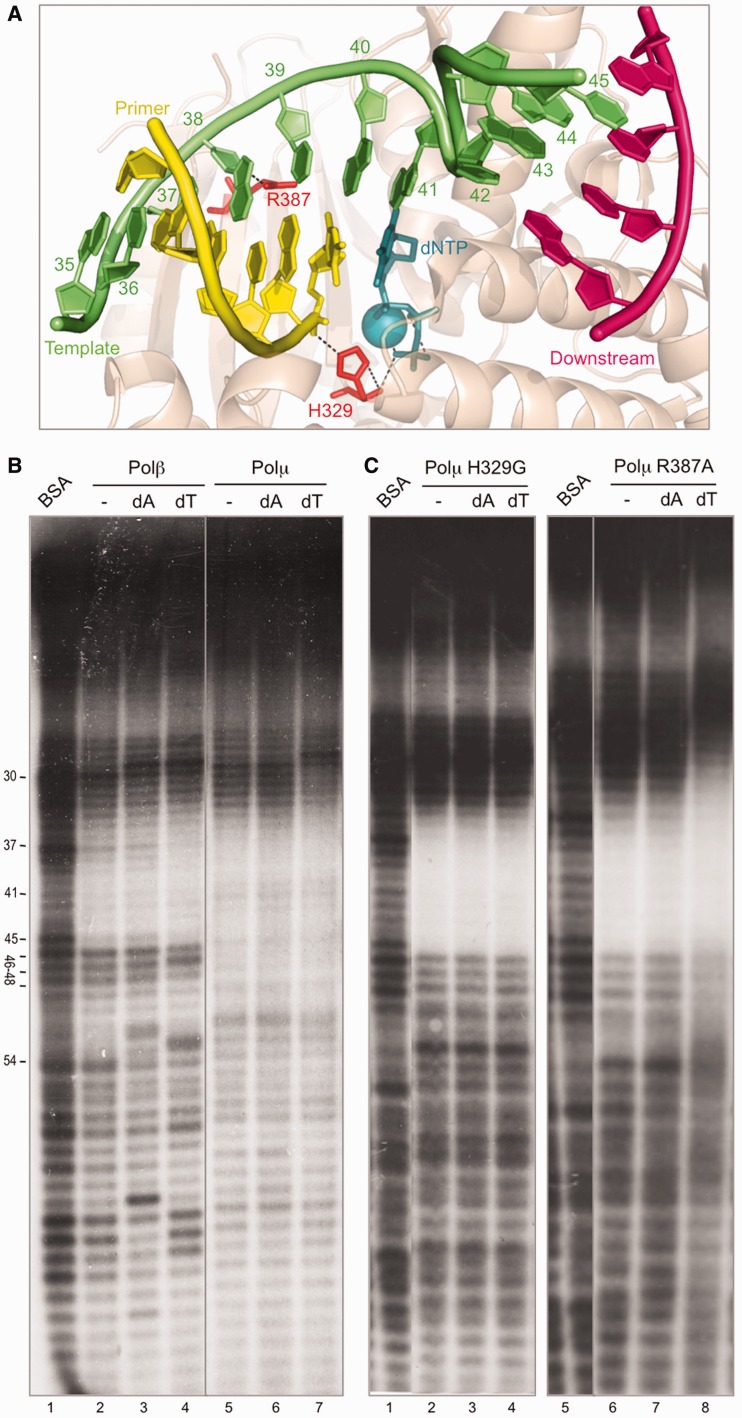
Ternary complex formation in solution. (**A**) Structure of Polµ (shown in wheat-colored ribbons) ternary complex (2IHM), in which the DNA substrate and incoming nucleotide are shown in sticks with the following colors: dNTP, dark teal; template strand, green; primer strand, yellow; downstream strand, dark pink. Selected residues are shown in red sticks. Nucleotides in the template strand are numbered as in the footprinting assays for clarity. (**B**) Footprinting assay of the wild-type Polβ (1.5 µg) and Polµ (1.5 µg). When indicated, 100 µM dATP or dTTP were added, together with 2.5 mM MgCl_2_. The DNA substrate used, formed by hybridizing the oligonucleotides FP-T (template, labeled at its 5′-end), FP-P (primer) and FP-D (downstream) always contains a phosphate group at the 5′-end of the downstream strand. Gel was dried and the labeled fragments detected by autoradiography. (**C**) Footprinting assays of Polµ mutants H329G and R387K (1.5 µg) were carried out as described in (B).

In the case of Polβ, addition of the complementary deoxynucleotide (dT), but not the non-complementary (dA), enhanced the footprint of the polymerase (see a lighter band at position 37) and expanded further into the primer zone (Figure 2B, compare lanes 2–4 up to position 33), indicative of a subtle adjustment of the polymerase conformation, allowing a stronger binding to the primer region. Upon addition of either deoxynucleotide, a new hypersensitivity appears at position 29. It should be noted that there was an initial hypersensitivity at position 30, i.e. frequent cuts or nicks are produced by DNAseI at this position in the DNA substrate. These nicks are an ideal substrate for Polβ, since they mimic the intermediate substrates of the BER pathway in which this polymerase is implicated. As the footprint is being carried out in the presence of activating metal ions (Mg^2+^), Polβ would insert a deoxynucleotide on this nicked substrate, leading to the formation of a +1 product that appears at position 29. Changes at other bands can be also explained as polymerization events, not related to the specific footprint centered at the gap (position 41).

Also in the case of Polµ, the area protected by the polymerase was slightly extended only upon addition of the correct deoxynucleotide (Figure 2B, lane 7), indicating that the complementary deoxynucleotide promotes an adjustment that allows a better binding to the primer area. On the other hand, we can see the appearance of the hypersensitivity at position 29, but unlike what happened with Polβ, the hypersensitivity only changed from position 30 to 29 when adding the complementary nucleotide (Figure 2B, lane 7), and not with the non-complementary one (lane 6).

To confirm that the extension of the footprint was in fact due to the interaction with the nucleotide, we used two mutants of Polµ (27): H329G (eliminating the function of a residue involved in nucleotide binding; Figure 2A) and R387A (mutating not a nucleotide ligand, but a dual [template/primer] DNA ligand, thus allowing ternary complex formation; Figure 2A). As expected, the footprint obtained with mutant H329G does not expand with the addition of nucleotide, either correct or incorrect (Figure 2C, lanes 3 and 4), while the footprint produced by mutant R387A, which itself is able to bind the nucleotide, is clearly expanded to the primer side upon addition of the correct dTTP (Figure 2C, lane 8). Strikingly, these mutants do not display the downstream expansion of the footprint probably corresponding to the BRCT domain, and this could be due to their inability to form a correct binary complex with the DNA substrate. That defect was partially recovered only in the case of mutant R387A upon addition of the correct (dT) nucleotide (Figure 2C, lane 8).

### Intrinsic Polµ binding to NHEJ substrates

**Figure 3.**
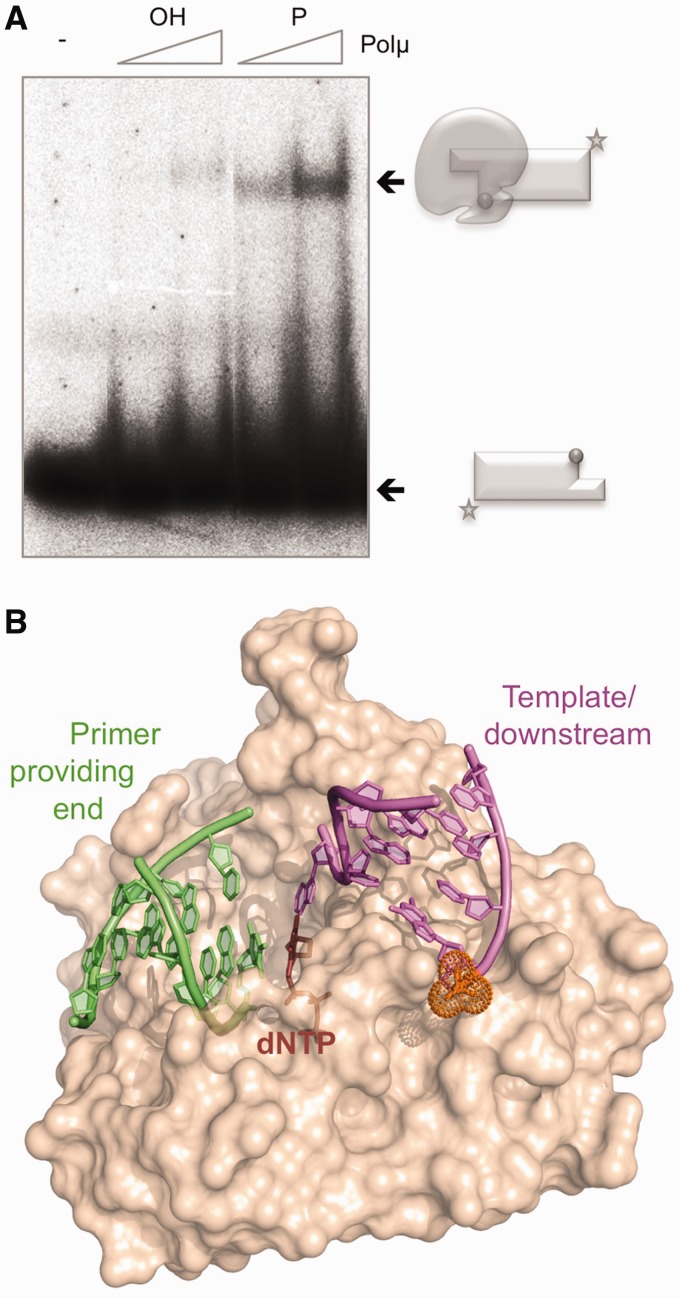
Intrinsic Polµ binding to NHEJ substrates. (**A**) EMSA of Polµ (300 and 600 nM) using T/D substrates (formed by hybridization of GT and NHEJ-D oligonucleotides) either lacking (OH) or having (P) a phosphate group at the 5′-end of the downstream strand. The schemes show the free substrate (with a sphere depicting the 5′-P group) or the polymerase bound to the substrate. (**B**) Schematic representation of the possible arrangement of the three NHEJ substrates (the two DNA ends and the incoming nucleotide) mediated by Polµ.

Polµ has been implicated in the NHEJ pathway of DSB repair (9,10,12,16,28). This DNA repair mechanism implies bridging of two DNA termini that can be either blunt or 5′-/3′-protruding. In the case of 5′-protruding ends, DNA synthesis can occur in a step prior to end-bridging due to the simultaneous presence of a recessive 3′-hydroxyl group and a template strand, both being requisites for most DNA polymerases, and the resulting blunt ends could be directly ligated. Conversely, polymerization on a 3′-protruding end requires either terminal transferase activity or the previous connection of the two ends to provide a template strand. The ability to bind this kind of 3′-protruding DNA substrates by a DNA polymerase would imply an advantage for filling in the gaps formed during the end-joining process. However, binding to these molecules could occur in two ways, since the DNA polymerase could recognize the 3′-protrusion either as the 3′-primer terminus or as a template strand. To elucidate the binding mode of Polµ to these substrates, 3′-protruding molecules either bearing or lacking a 5′-P group in the recessive strand were tested. In order to minimize any possible self-pairing, allowed by molecules containing very long single-stranded portions, we used a set of molecules with very short (3 nt) 3′-protruding ends. EMSA demonstrated that Polµ is able to form a 3-fold more stable complex (also with a slightly higher mobility) when binding 5′-P-containing 3′-protruding substrates (Figure 3A, KD values are 400 nM for the 5′-P-lacking versus 165 nM for the 5′-P-containing substrate). Considering the previous results using gapped substrates (Figure 1), and the recognition of the 5′-phosphate by the 8-kDa domain, it is very likely that the presence of the 5′-P orients binding, thus selecting the 3′-protrusion as a template strand (see scheme). When the phosphate is not present, a much weaker binding to the 3′-protrusion is occurring, now likely oriented as a primer strand. This specific binding, reinforced by the 5′-P, would help Polµ to initiate bridging of two DNA ends (a 3′-protrusion is firstly recognized as the template-providing end, and the 3′-protrusion of a second end must then be recognized as primer) during the NHEJ reaction (modeled in Figure 3B).

### Impact of Polµ DNA-binding properties on its enzymatic activity during NHEJ

**Figure 4.**
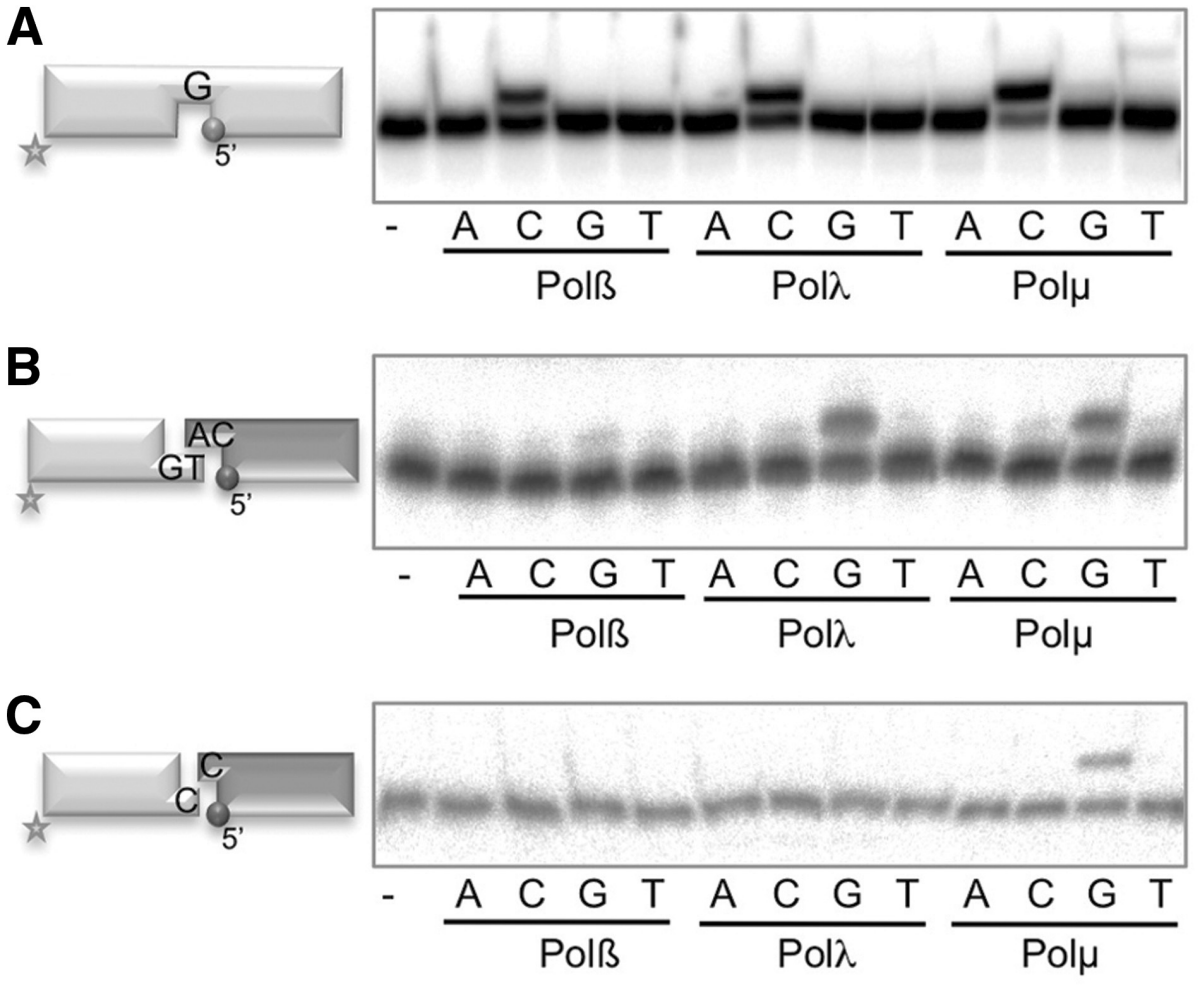
Impact of Polµ DNA-binding properties on its enzymatic activity during NHEJ. (**A**) Gap-filling activity of Polβ, Polλ and Polµ (25 nM each) was assayed using a substrate formed by the hybridization of the oligonucleotides SP1C, T28 and D12. When indicated, 10 nM of each dNTP was added, in the presence of 2.5 mM MgCl_2_. (**B**) NHEJ assay of Polβ (600 nM), Polλ (600 nM) and Polµ (200 nM) was performed as described in ‘Materials and Methods’ section, using a set of compatible substrates: the labeled substrate was formed by hybridization of GT and NHEJ-D (shown in light gray) and the cold substrate by hybridization of CA and NHEJ-D (shown in dark gray). When indicated, dNTPs were added separately at 100 µM in the presence of 1 mM MnCl_2_ for Polβ and Polλ, and 2.5 mM MgCl_2_ for Polµ. After electrophoresis, the labeled fragments were detected by autoradiography. (**C**) NHEJ reaction performed as in (B), with a set of incompatible substrates in which both the labeled (light gray) and cold (dark gray) molecules were formed by hybridization of C- and D-NHEJ. When indicated, the substrates contain a 5′-P group at the downstream strand (dark gray spheres).

In agreement with their similar Polβ-like core, the three template-directed polymerases from the human X family, i.e. Polβ, Polλ and Polµ, are similarly competent when polymerizing on a small gap (as shown in Figure 4A). Conversely, it has been shown that Polβ is not a player of the NHEJ pathway, while Polλ and Polµ, having a BRCT domain that interacts with NHEJ core factors, are the main polymerases implicated; moreover, Polλ and Polµ are not redundant since in the presence of NHEJ accessory factors such as Ku70/80 and XRCC4/LigaseIV, both of them can use complementary ends but only Polµ is able to join non-complementary ends (11). To evaluate if these differences are intrinsic to each enzyme, we tested the three polymerases Polβ, Polλ and Polµ, in NHEJ *in vitro* reactions in which the polymerase alone, in the absence of NHEJ accessory factors, is challenged to bridge two DNA ends, extending one 3′-end by copying the templating base provided by a second 3′-end. For that we used two sets of short double-stranded DNA molecules: one set containing a 3′-overhang of 2 nt which can form 1 bp (Figure 4B, scheme), and the other set of DNA ends, whose 1-nt 3′-protrusion (dC) provides null complementarity when confronted to each other (Figure 4C, scheme). In the two cases, one DNA end (depicted in light gray) was labeled at the 5′-terminus of the 3′-protruding strand, and therefore can be assayed for primer extension, whereas the second molecule (depicted in dark gray), unlabeled, provides the templating base (dC) *in trans.* Thus, preferential extension of the primer with ddG (having the same affinity as dG in all PolXs, but used to avoid any extension further to +1) would be indicative of an accurate first stage of NHEJ. When working with DNA substrates bearing complementary 3′-protruding ends, we observed that, as expected, Polβ had very little activity, while Polλ and Polµ can efficiently and accurately insert the correct nucleotide (ddG) dictated by the templating base (dC) provided *in trans* by the second end (Figure 4B). On the other hand, when using DNA substrates that did not contain any microhomology, both Polβ and Polλ were inactive and only Polµ was able to perform an efficient and accurate NHEJ reaction (Figure 4C), a similar behavior shown to occur at some incompatible ends when Polµ was assisted by NHEJ core factors (29). Therefore, our results allow to conclude that the capacity of Polµ to perform NHEJ of incompatible ends is an intrinsic property of the enzyme, not conferred by additional NHEJ protein factors.

### Importance of 5′-P recognition for Polµ-mediated NHEJ

**Figure 5.**
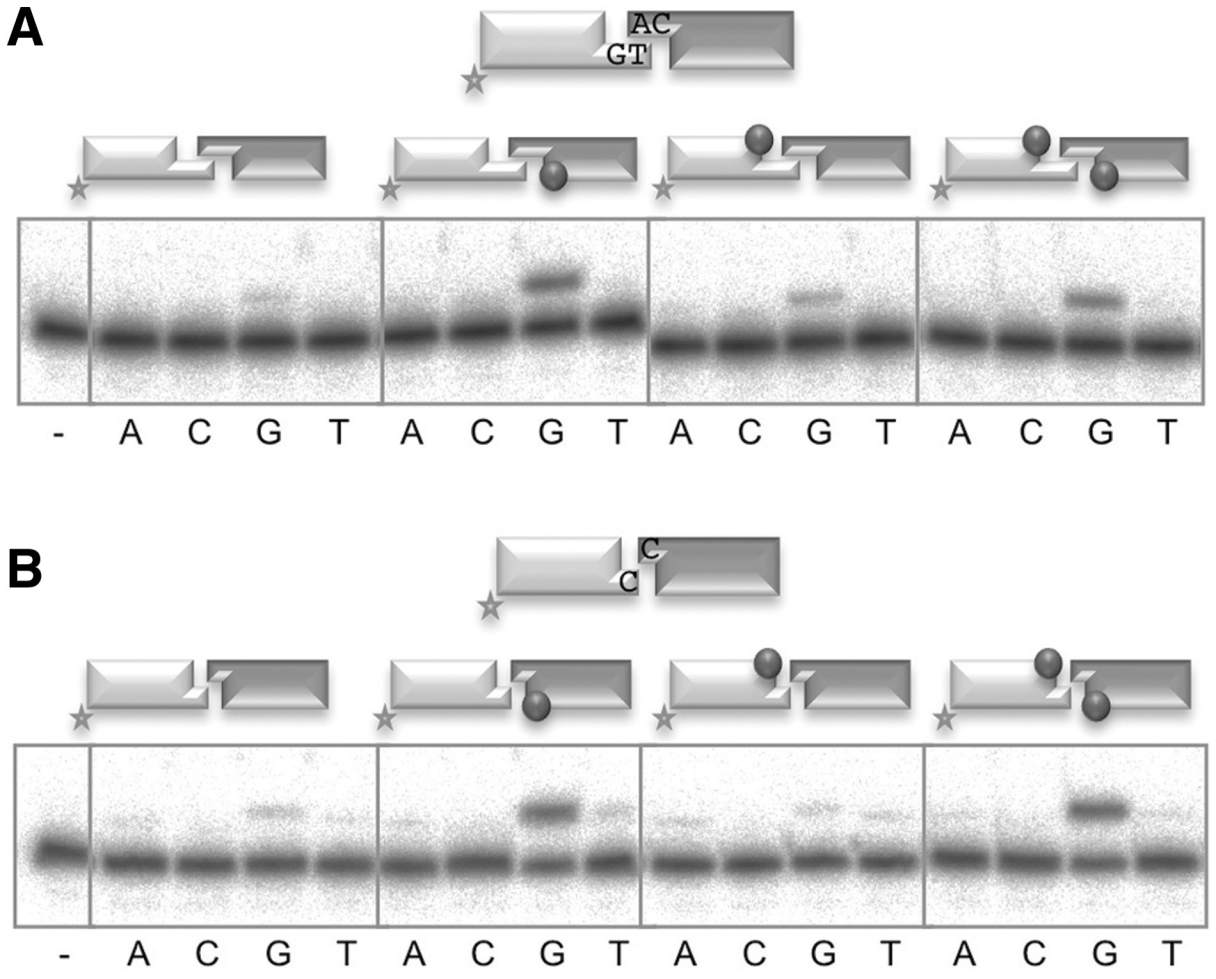
Importance of 5′-P recognition for Polµ-mediated NHEJ. (**A**) NHEJ assay of Polµ (200 nM) performed as described in ‘Materials and Methods’ section, using a set of compatible substrates: the labeled substrate was formed by hybridization of GT and NHEJ-D (light gray) and the cold substrate by hybridization of CA and NHEJ-D (dark gray). The dark gray spheres indicate the presence of a 5′-P group in the downstream strand of the substrate. When indicated, dNTPs were added separately at 1 µM in the presence of 2.5 mM MgCl_2_. After electrophoresis, the labeled fragments were detected by autoradiography. (**B**) NHEJ reaction performed as in (**C**), with a set of incompatible substrates in which both the labeled (light gray) and cold (dark gray) molecules were formed by hybridization of C and NHEJ-D.

As shown here, the presence of a downstream 5′-P group is important for Polµ binding to NHEJ substrates. However, and in spite of being very early anticipated (12), the impact of this requirement on the activity of family X polymerases during NHEJ has not been directly evaluated. So, we first tested if the presence of a 5′-P in either or both of the two DNA ends was required for Polµ bridging activity during NHEJ, by using the same kind of complementary and non-complementary pairs of molecules as in Figure 4. The results showed that the 5′-P of the template-providing end (dark gray) is essential for an efficient and accurate nucleotide insertion, on both compatible (Figure 5A, compare first and second panels) and incompatible (Figure 5B, compare first and second panels) substrates; conversely, the presence of the 5′-P in the primer-providing end (light gray), flanking a second gap, is unnecessary (Figure 5A, compare first versus third panels; Figure 5A and B, compare second versus fourth panels), and in some cases even detrimental for this activity (Figure 5B, compare first and third panels). A plausible explanation, in agreement with the EMSA experiments described in the previous section, is that a recessive 5′-P only in the labeled DNA end (light gray) would orient enzyme recognition in such a way that the labeled molecule would be preferentially selected to act as template (strongly competing with the cold one) instead of acting as primer.

### NHEJ-specific Polµ residues acting as ligands of the priming end

**Figure 6.**
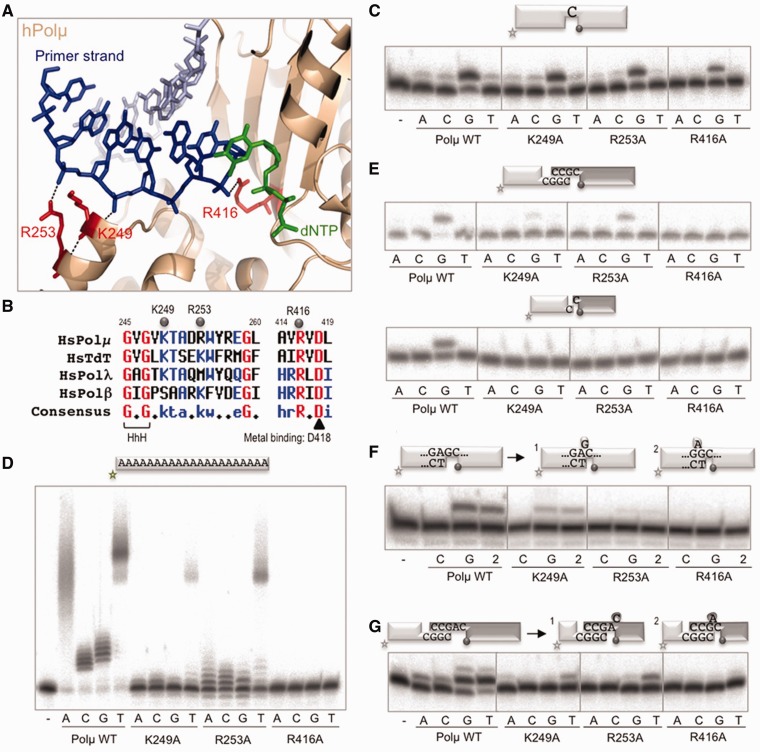
NHEJ-specific Polµ residues acting as ligands of the priming end. (A) Cartoon representation of the ternary complex structure of Polµ (PDB ID: 2IHM), in which the DNA substrate has been modified to mimic a 1-nt 3′-protruding substrate in a template/primer orientation, colored light/dark blue. The incoming dNTP is shown in green. The residues selected for mutagenesis are shown in red sticks, and the polar contacts established with the primer strand are highlighted in black. (**B**) Sequence alignment of the four X family members in humans, showing the two regions in which the mutations were made. Numbering indicates the residues in the Polµ sequence. Mutated residues are indicated with gray dots. (**C**) Gap-filling reactions were performed as described in ‘Materials and Methods’ section with the indicated proteins (25 nM) using a gapped substrate containing the oligonucleotides SP1C, T28 and D12. When indicated, dNTPs were added separately at 10 nM in the presence of 2.5 mM MgCl_2_. (**D**) Terminal transferase activity assay with the indicated proteins (600 nM) using a homopolymeric substrate (polydA) and each of the four dNTPs (100 µM). Reactions were performed for 30 min at 37°C. The insertion of dTTP which can be still observed with the mutants is not a strict terminal transferase reaction, but the result of connecting two ssDNA substrates and subsequently copying the polydA template. (**E**) NHEJ reactions were performed with 200 nM of the indicated proteins and using four sets of substrates: the labeled substrates were formed by hybridization of C with NHEJ-D or D3 with D1, and the cold substrates, by hybridization of either C with NHEJ-D or D4 with D2. The dark gray spheres indicate the presence of a 5′-P group in the downstream strand of the substrate. When indicated, each of the four ddNTPs (10 µM) were added in the presence of 2.5 mM MgCl_2_. (**F**) Gap-filling reactions were performed as in (C) but using a 2-nt gapped substrate containing the oligonucleotides P15, T32 and D16. When indicated, the dNTP complementary to the first (dCTP), second (dGTP) or both templating bases were added (10 nM). (**G**) NHEJ reactions were performed as in (E) but using one set of substrates that gives shape to a 2-nt gap once joined: the labeled substrate contained the oligonucleotides D3 and D1, and the cold substrate, D4–AC and D2. The dark gray spheres indicate the presence of a 5′-P group in the downstream strand of the substrate. When indicated, each of the four dNTPs (10 µM) was added in the presence of 2.5 mM MgCl_2_.

During NHEJ of 3′-protruding ends, Polµ has to be able to interact with three substrates at once, i.e. the incoming nucleotide and the two DNA ends that need to be joined. In order to maintain all the substrates in proper register for polymerization to take place, Polµ has to maintain a number of interactions with each of them. We have already established the direct impact of the 5′-P group on the maintenance of a firm grip of the downstream side of the break, and the importance for NHEJ of one specific residue involved in the dual interaction with the incoming nucleotide and the 3′-terminus of the primer, His^329^, has been documented (27,30). Further inspection of the contacts between the polymerase and the DNA led us to focus on three other residues in Polµ that interact with the primer strand: Lys^249^, Arg^253^ and Arg^416^, as shown in Figure 6A (Supplementary Figure S2A). Figure 6B shows the level of conservation of each of these three residues among the members of the X family: the positive charge of Lys^249^ that contacts the −4 position of the primer, is conserved in the other two NHEJ-related polymerases, Polµ and TdT, but not in the case of Polβ in which a serine occupies this position; in the case of Arg^253^, which interacts with the −5 position of the primer (flanking the upstream side of the footprint), a similar contact (via a lysine) is maintained in Polβ and TdT, but not in Polλ that has a methionine; Arg^416^ that interacts with the 3′-terminus of the primer, is completely invariant in all the members of the family.

We mutated each of them to alanine in order to abolish their function. None of the individual mutations significantly affected gap-filling, with the exception of R416A that showed a 40% reduction in activity (Figure 6C). On the other hand, when these mutants were tested for terminal transferase activity, all of them showed a strong reduction in the level of untemplated incorporations (Figure 6D). This phenotype was expected assuming that these ligands are instrumental to bind the primer strand and orient the 3′-terminus for catalysis, thus being essential in the absence of a template strand and a stabilizing 5′-P. When we tested these mutants for NHEJ activity, all of the mutations completely abolished activity on non-complementary ends (Figure 6E, bottom panels), but both K249A and R253A maintained a certain level of activity on complementary substrates (Figure 6E, top panels). These results demonstrate the importance of these residues for Polµ during NHEJ, particularly for the most difficult task of connecting non-compatible ends.

Thus, Polµ is well suited for joining two ends during DNA repair reactions because it simultaneously binds both the 5′- and 3′-termini of the gap. These very specific binding properties, however, could be inconvenient during the filling-in of longer gaps: due to the very close location of the 3′-OH and 5′-P in the active site, the yet-to-be-copied nucleotides in the template strand necessarily have to be held in a ‘bubble’ until they can be used. In fact, the first crystal structure of Polλ, which included the polymerase in an inactive binary complex with a 2-nt gap (31), suggested that in the absence of an incoming nucleotide, the polymerase might preferentially bind the 5′-end of the gap without correctly engaging the 3′-primer terminus, a feature that most surely applies to Polµ as well (Supplementary Figure S2B, left scheme). In a more recent structure of the catalytically active ternary complex of Polλ bound to a 2-nt gap (32), a conformational change has occurred, allowing the polymerase active site to bind the 3′-primer terminus while the 8-kDa domain remains bound to the 5′-end of the gap. For this to happen, the template strand has to be ‘scrunched’, and the second templating nucleotide flipped-out. In the case of family X polymerases, this bubble of flipped-out nucleotides in the template strand can be positioned in at least two different locations: (i) in the ‘scrunching’ pocket downstream to the polymerization site (Supplementary Figure S2B, scheme 1), as in the case of Polλ, where the gap is correctly filled-in step by step; (ii) upstream to the polymerization site via template dislocation (Supplementary Figure S2B, scheme 2), thus implying loss of sequence and generation of frameshifts since these templating nucleotides will not be copied but left behind. In the case of Polµ, frameshift generation can be further implemented if the 3′-primer terminus can be realigned with the +1 position of the template [‘slippage’ mechanism, described initially for Polβ in (33), see Supplementary Figure S2B, scheme 3].

Given the importance of Lys^249^, Arg^253^ and Arg^416^ in stabilizing the primer strand, we decided to test the mutants for polymerization on a 2-nt gapped substrate. Regarding template selection, mutants behaved like the wild-type Polµ, incorporating preferentially the nucleotide (dGTP) complementary to the second templating base (dC), and producing π1 frameshifts even in the presence of the 2 nt needed to correctly fill the gap (Figure 6F). These results are in agreement with the strong binding of Polµ to the 5′-P that likely triggers a ‘dNTP selection’-mediated dislocation in which the polymerase produces a 1-nt gap by flipping-out the first templating nucleotide (see scheme 1 in Figure 6F). In the case of the sequence used here, this same result can also be achieved by flipping out the −1 nt, since the primer can be realigned to form a favorable/stable T:G mismatch (scheme 2) In spite of making similar template dislocations, the activity level of the mutants was strongly affected, specially for R253A and R416A, Thus, the lack of any of these positively charged residues could affect the 2-nt gap filling reaction by reducing Polµ’s capacity to pull the primer strand (generating the dislocation of the template) neighbor to the nucleotide site. This site is contiguous to the 5′-P of the downstream strand, the most important DNA-binding constraint for this polymerase, either when filling-in a gap, or, more importantly, during the first step of NHEJ. To test this last hypothesis, we assayed the activity of the mutants on complementary NHEJ substrates that once joined lead to the formation of a 2-nt gap (Figure 6G, see scheme). In this context, wild-type Polµ was able to extend the primer either with dTTP, copying the first templating base (dA, with dC in the ‘scrunching’ pocket as in scheme 1), or with dGTP, directly copying the second (dC, flipping out dA, as in scheme 2). We tested a range of other possible sequence contexts producing 2-nt gaps during a NHEJ reaction, and our results showed that Poµ dislocates the template strand producing a mutagenic outcome in a majority of the cases (Supplementary Figure S3). As expected, mutant R416A was unable to polymerize on this kind of substrates. The activity of mutants K249A and R253A was strongly affected but detectable and, strikingly, only dT was inserted, meaning that the presence of these residues is what renders Polµ able to promote the flipping-out of the templating dA (Figure 6G, scheme 2), and thus, to dislocate the template strand producing −1 frameshifts in a NHEJ context. Thus, elimination of any of these charged residues (Lys^249^ or Arg^253^) transforms Polµ into a Polλ-like polymerase, which will produce a less mutagenic outcome during NHEJ by promoting only the ‘scrunching’ solution, which allows correct counting and gap filling. The downside of such a polymerase, however, would be a considerable loss in efficiency, an effect appropriately avoided in Polµ.

**Figure 7.**
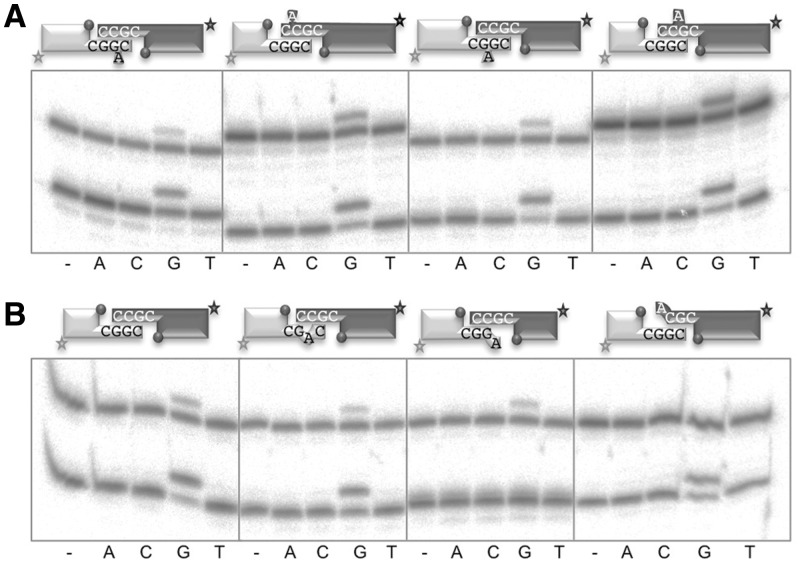
Polµ-mediated NHEJ: non-aligned ends. (**A**) NHEJ reactions performed as described in ‘Materials and Methods’ section, with 200 nM Polµ and using labeled compatible substrates: the short, fast running substrates were formed by hybridization of either D3 (second and fourth schemes), D3BB1 (first scheme) or D3BB2 (third scheme) with D1 (shown in light gray) and the long, slow running substrates, by hybridization of either D4 (first and third schemes), D4BB1 (second scheme) or D4BB2 (fourth scheme) with D2 (shown in dark gray). The dark gray spheres indicate the presence of a 5′-P group in the downstream strand of the substrate. When indicated, dNTPs were added separately at 1 µM in the presence of 2.5 mM MgCl_2_. After electrophoresis, the labeled fragments were detected by autoradiography. (**B**) NHEJ reactions performed as in (A), using labeled compatible substrates: the short substrates were formed by hybridization of either D3 (second and fourth schemes), D3MM (first scheme) or D3FLAP (third scheme) with D1 (shown in dark gray) and the long substrates, by hybridization of either D4 (first to third schemes) or D4FLAP (fourth scheme) or D4BB2 (fourth scheme) with D2 (shown in dark gray). The dark gray spheres indicate the presence of a 5′-P group in the downstream strand of the substrate.

The importance of these residues during Polµ-mediated NHEJ is congruent with: first, the non-continuous nature of the NHEJ substrates that implies the need for a tighter binding of the primer strand; second, the highly probable formation of gaps >1 nt and also distortions in the connection, such as mismatches or flipped-out nucleotides, 3′-flaps of ssDNA, etc., that need to be accepted by the polymerase (34). To assess NHEJ efficiency when these imperfections are present at a Polµ-mediated synapsis, we used a set of NHEJ substrates with variable protrusions, able to form 2 or 3 bp of complementarity, but with a range of distortions or mismatches in the synapsis. The results, corresponding to the simultaneous evaluation of NHEJ reactions carried out at both gaps flanking the synapsis, showed that Polµ can deal with complementary ends containing 1-nt ‘bubbles’ at the − 1 and − 2 positions of each primer (Figure 7A) with the same efficiency as with perfectly complementary ends (Figure 7B, first panel). We confirmed this result by testing the four different options as template to make sure that the synapsis was being correctly formed (Supplementary Figure S4). The presence of a mismatch in the connection does not seem to have any deleterious effect either (Figure 7B, second panel). However, the presence of a 1-nt 3′-flap (a mismatched 3′-primer terminus) totally inhibits polymerization on that DNA end, but not on the other (Figure 7B, third and fourth panels).

In conclusion, Lys^249^, Arg^253^ and Arg^416^ render Polµ into a more mutagenic polymerase, but at the same time allow a tighter binding of the primer strand, a property which is particularly required during exceptionally challenging NHEJ reactions such as the ones extensively studied here.

### The BRCT domain of Polµ contributes, via DNA binding, to its intrinsic ability of joining DNA ends

**Figure 8.**
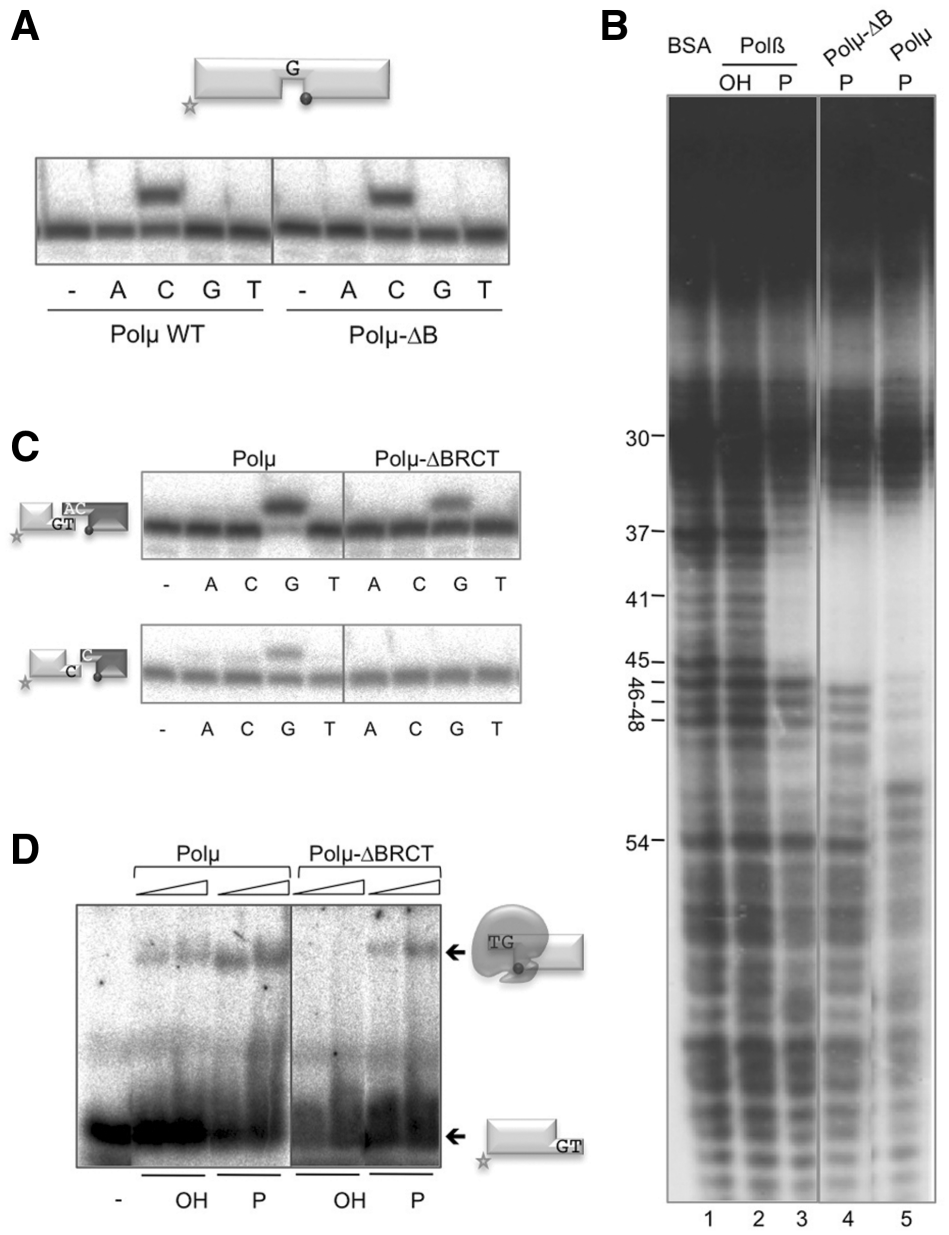
The BRCT domain of Polµ contributes, *via* DNA binding, to its intrinsic ability of joining DNA ends. (**A**) Gap-filling reactions were performed as described in ‘Materials and Methods’ section for the indicated proteins (25 nM) using a gapped substrate containing the oligonucleotides SP1C, T28 and D12. When indicated, dNTPs were added separately at 10 nM in the presence of 2.5 mM MgCl_2_. (**B**) Footprinting assay of the wild-type Polβ (1.5 µg) and mutant and wild-type Polµ (1.5 µg). The DNA substrate used, formed by hybridization of the oligonucleotides FP-T (template, labeled at its 5′-end), FP-P (primer) and FP-D (downstream) may have (P) or lack (OH) a phosphate group at the 5′-end of the downstream strand. (**C**) NHEJ reactions were performed as described in ‘Materials and Methods’ section, with 200 nM Polµ and using two sets of substrates: the labeled substrates were formed by hybridization of GT or C with NHEJ-D, and the cold substrates, by hybridization of either CA or C with NHEJ-D. The dark gray spheres indicate the presence of a 5′-P group in the downstream strand of the substrate. When indicated, dNTPs were added separately at 10 µM in the presence of 2.5 mM MgCl_2_. The efficiency of the polymerization reaction is defined as a function of the percentage of primer extension. We used two different software programs to obtain the measurement of the extended primer in a minimum of three different replica experiments. (**D**) EMSA was performed with the indicated proteins (300 and 600 nM) using a 3′-protruding substrate containing the oligonucleotides GT and NHEJ-D.

We have shown that Polµ is able to join two DNA ends in the absence of any NHEJ accessory factors such as Ku or XRCC4/LigIV [(27); Figure 4], to which Polµ interacts via its BRCT domain (15), suggesting that Polµ could participate in the alternative end-joining pathway that takes place without the need for the classic NHEJ factors. To test if this activity is inherent to the enzyme core, we tested if the Polβ-like polymerization domain of Polµ was able to perform DNA end-bridging and *trans*-directed polymerization. For this, we prepared a deletion mutant lacking the BRCT domain (Polµ-ΔBRCT; see ‘Materials and Methods’ section) and confirmed, as a control, that its gap-filling activity (both efficiency and fidelity) were comparable to those of the wild-type enzyme (Figure 8A). DNAseI footprinting experiments on a DNA gap revealed a protection caused by the Polµ core (Figure 8B, lane 4) which spans a total of 10 nt of the template strand: five involved in base pairing the primer strand, one free base (the templating nucleotide in the gap; number 41) and four bases paired to the downstream strand. This protection matches that obtained with Polβ on the 5′-P-containing gap (Figure 8B, compare lanes 2 and 3). Moreover, these footprints are fully compatible with the 3D structural information available for the ternary complexes of both DNA polymerases (PDB IDs: 2IHM for Polµ and 1BPY for Polβ; Supplementary Figure S1). As already noted, by using the full-length (i.e. BRCT-containing) Polµ, the footprint is further extended (6 nt) toward the downstream strand (Figure 8B, lane 5), thus confirming that this extension is due to the intrinsic binding of the BRCT domain, downstream to the core, to a gapped DNA substrate.

**Figure 9.**
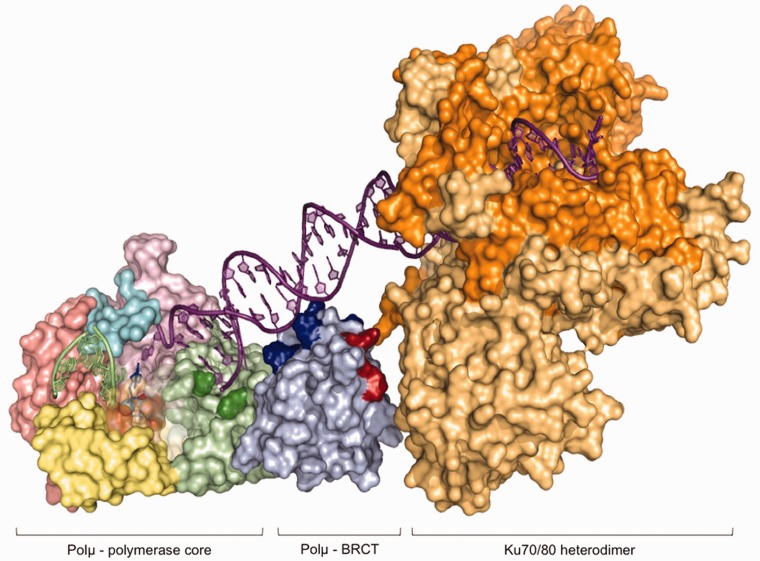
Model of the interaction of Polµ with the Ku heterodimer and the DNA substrate through the BRCT domain. Computer-generated model of the predicted location of the Polµ β-like core, the BRCT domain and the Ku70/80 heterodimer on a single DNA end. The different domains in the Polµ core have been colored as follows: 8-kDa domain in green (with the residues forming the 5′-P pocket highlighted in dark green), the fingers subdomain in yellow, the palm subdomain in salmon, the thumb subdomain in pink and the Loop1 motif in cyan. The BRCT domain is colored light blue, with the residues predicted to be interacting with the DNA substrate in dark blue and the residues implicated in the interaction with the NHEJ factors in red. The two subunits of the Ku heterodimer are colored in dark and light orange.

It can be assumed that a similar BRCT/DNA interaction occurs during NHEJ of both complementary and non-complementary ends, being restricted to the DNA end that provides the template (the downstream side of the break), thus reinforcing the interaction already mediated by the 5′-P. Accordingly, the NHEJ activity of the Polµ-ΔBRCT mutant was at least 3-fold less efficient (as indicated by the percentage of primer extended) than that of the full-length enzyme, particularly when joining incompatible ends, on which the activity of the Polµ core was undetectable (Figure 8C, lower panels). These results suggest that the BRCT domain of Polµ could benefit NHEJ by promoting a tighter binding of Polµ to the DNA substrates. To corroborate this hypothesis, we tested both proteins in EMSA of a short 3′-protruding (GT) DNA end having or lacking a recessed 5′-P. Whereas the full-length Polµ produced a stable retarded band even in the absence of a 5′-P, Polµ-ΔBRCT binding to DNA was much weaker (KD of 2 µM), being undetectable in the absence of a 5′-P (Figure 8D). The BRCT domains of Polµ, Polλ and TdT have been crystallized, but independently from the corresponding polymerase cores. This lack of combined structural information hampered the prediction about whether this domain could be involved in protein–DNA interactions in any of these polymerases, besides its function as a protein–protein interaction domain (11,14). As a result of our experimental data and helped by 3D modeling, the simultaneous interaction of Polµ BRCT with both the DNA substrate (through its downstream part) and with the Ku70/80 heterodimer (Figure 9) will be described in the ‘Discussion’ section.

## DISCUSSION

### Polµ in NHEJ: the dexterity of clutching two chains with one hand

The NHEJ pathway takes advantage of highly-specialized polymerases, capable of dealing with two DNA ends at once: one providing a broken primer strand with a protruding 3′-OH, and a second end that provides a recessive 5′-P, and a broken template strand, also 3′-protruding. Thus, for this task, two different pieces of DNA need to be hold by the same enzyme molecule. Compelling evidence indicates that the NHEJ pathway minimizes loss of genetic material by using any template available: the overhangs formed during alignment of DSBs are usually filled in, allowing retention of the maximum sequence possible (3,35). In this context, a NHEJ polymerase must use a primer provided by one end and a template provided *in trans* by a second end: ‘alignment-based gap fill-in’. In the case of X family polymerases, the ability to act during NHEJ is dictated by their template dependence, a property that follows a gradient ranging from Polβ that only polymerizes on substrates with a continuous template strand; to Polλ that is active in NHEJ only when the template strand is stabilized by complementarity with the primer strand; to Polµ, who can direct template-dependent synthesis even when there is no base pairing between the two ends; to TdT that also acts on unpaired primer termini but does not allow the use of a template strand [reviewed in (16)]. It has been suggested that this variable degree of template dependence relies on structural differences among the four polymerases, such as Loop1, exclusively present in TdT and Polµ (11,17,36). In this article, we have studied several structural DNA-binding determinants, in particular those conferring Polµ the unique handiness of *trans-*polymerizing without the help of a single connecting base pair.

PolXs have intrinsic capacities of gap recognition and binding involving simultaneous recognition of both sides of the gap. Such a dual DNA binding is more crucial for Polλ and Polµ, polymerases not as specialized as Polβ in using substrates with a continuous template strand (i.e. gaps), but also in charge of bridging two separate DNA ends. The ability to independently bind and orient two DNA ends is thus closely related to their function during NHEJ. In order to bind the upstream side of the gap, an invariant arginine residue of the palm subdomain (Arg^416^ in Polµ) interacts with the primer, positioning the 3′-terminus next to the nucleotide-binding site; a second residue contributing to this positioning is only conserved in Polµ and TdT [His^329^/His^342^; (30,36)]; moreover, the fingers domain in X family polymerases provides a primer-binding platform which interacts with the upstream duplex through a helix–hairpin–helix (HhH) motif formed by α-helices F and G. In Polµ, as well as in TdT, the contacts between this HhH motif and the upstream primer are more numerous than those found in Polβ and Polλ, probably allowing for the stronger primer-binding needed for terminal transferase or NHEJ reactions involving little or null complementarity. That hypothesis has been proven here by mutating Polµ residues Lys^249^, Arg^253^ (HhH motif), Arg^416^ and His^329^. As expected, all of these mutations affected specifically the reactions in which a template strand is discontinuous: terminal transferase and end-joining, while not affecting 1-nt gap-filling (Arg^416^, invariant in all X family members, was shown to be of general importance for polymerization).

The defective polymerization of these mutants on longer gaps, indicates their key role in ‘pulling’ the primer strand and bringing it closer to the 5′-P, while at the same time producing a ‘bubble’ in the template strand. The level of conservation of these residues, being all of them present only in Polµ while Polλ and Polβ have lost some of these contacts, alludes to their importance for Polµ-specific activities that are not present in the other enzymes, namely the ability to carry out untemplated additions and also the exceptional talent of bridging and executing template-directed insertions on two non-complementary DNA ends.

The unique structural feature that allows polymerases from X family to bind the downstream side of gapped and NHEJ substrates is the 8-kDa domain. In the ternary structures of Polβ (PDB ID: 1BPY), Polλ (PDB ID: 1XSN) and Polµ (PDB ID: 2IHM), the 5′-P moiety is located at a positively charged pocket in this domain, where binding is mediated by several hydrogen bonding interactions with basic side chains. The 8-kDa domain contains another structural motif implicated in DNA binding, the HhH motif. In Polβ, Polλ and Polµ structures, this HhH interacts with the downstream part of the substrate, suggesting that its function is the stabilization of the bent DNA, thereby facilitating the positioning of the two DNA ends in a NHEJ reaction. Therefore, the initial positioning of the polymerase domain in a gap (≥1 nt) is dictated by the binding of the 8-kDa domain to the 5′-P, and not by interactions with the primer terminus, which would occur in a second round of events. This conclusion has implications of great interest for the binding of the polymerase to NHEJ substrates, since the 8-kDa mediated binding would take place irrespective of the conformation of the 3′-end. The polymerase in charge for this then has to be able to take advantage of microhomologies for aligning the 3′-ends. The 8-kDa and the BRCT domains provide an initial anchoring point for this complicated task, and the primer-binding residues studied here are determinant for the difficult second step of bridging the two ends together.

### Role of the PolX BRCT domain in NHEJ

The members of the X family of polymerases are recruited to form a complex with the NHEJ core factors XRCC4/Ligase IV and Ku at the DNA break (11,14,37). Recent evidence has shown that BRCT domains can be specifically involved in the interaction with phospho-serine or phospho-threonine-containing motifs (38,39), an ability that may be involved in granting access of regulated proteins to the break, even though there is no evidence to date for a phosphorylation-dependent, BRCT-mediated, interaction of NHEJ factors.

Interestingly, sequence comparisons show that the BRCT of Polµ is most similar to TdT, with 39% sequence identity that includes the residues important for complex formation (40). This high level of sequence conservation is maintained at a 3D-structural level, in the BRCT domains of Polµ (PDB ID: 2DUN) and TdT (PDB ID: 2COE), as well as in their electrostatic surfaces, containing both a positively charged ridge on one face of the protein, and large negatively charged regions on the opposite faces. In the Polµ BRCT, the positive ridge is formed by Arg^44^, Arg^52^, Arg^85^ and Arg^86^ (Supplementary Figure S5, Patch I). This positive patch has been proposed to be involved in the interaction with a phospho-modified protein (40), but the results shown in this article suggest that a plausible function is the interaction with the downstream part of the DNA substrate: the complete lack of the domain already resulted in a diminished interaction with and activity on NHEJ substrates while not affecting other activities such as gap-filling. By orienting and overimposing the separate crystals of the BRCT domain and the Polµ core, we found out that one of the positive patches in the BRCT domain (Supplementary Figure S5, Patch I) perfectly accommodates the downstream part of the DNA substrate (Figure 9; colored in dark blue). We then modeled the interaction of the BRCT domain of Polµ with the Ku70/Ku80 heterodimer by orienting the DNA substrate. Strikingly, the side of the BRCT domain facing the Ku heterodimer in the model was exactly the one containing the residues putatively involved in this interaction (Figure 9; colored in red and Supplementary Figure S5, protein–protein interaction zone). According to this model, the portion of the DNA substrate which would be contacted by the BRCT domain flawlessly correlates with the length of the BRCT-specific protection (6 bp) observed in our footprinting assays.

Such a DNA-binding function of Polµ BRCT, independent of the core NHEJ factors, may enable this polymerase for a role in the loosely defined alternative end-joining pathway, which operates in the absence of classical NHEJ factors such as Ku, XRCC4 or DNA ligase IV. These repair events frequently involve small deletions and entail short stretches of homology at the break point (41–44). Polµ might bind the DNA break based on its own specificity for the 5′-P and through its BRCT domain and via its terminal transferase activity in charge of the additions that create the so-called polymerase-generated microhomology. In agreement with this proposed function, recent observations indicate that Polµ BRCT is atypical in the sense of not being involved in dimerization or multimerization (40). In fact, comparison of the structure of Polµ BRCT with other BRCT domains which effectively dimerize shows important differences, especially regarding R2 helix (45).

Taken together, all these different strategies and inventiveness found in a small enzyme, engineering several protein motifs to hold the DNA or other protein pieces of the polymerase in place for catalysis, and also the ambidexterity of the active site, prepared for almost any kind of DNA and nucleotide substrates as well as cofactors that need to be used, speak of Polµ as a highly specialized polymerase, singularly suited for its function: engagement of two different DNA chains for *trans-*directed polymerization, even in the case of inexistent complementarity, with the only objective of fulfilling the most efficient reaction possible, with the minimal loss of sequence, during NHEJ.

## SUPPLEMENTARY DATA

Supplementary Data are available at NAR Online: Supplementary Table 1 and Supplementary Figure 1–5.

## FUNDING

The Ministerio de Ciencia y Tecnologia Grant [BFU2009-10085 and CONSOLIDER CSD2007-00015]; an institutional grant to Centro de Biologia Molecular ‘Severo Ochoa’ from Fundacion Ramon Areces; fellowship from the Comunidad Autonoma de Madrid (to M.J.M.). Funding for open access charge: Ministerio de Ciencia y Tecnologia Grant [BFU2009-10085 and CONSOLIDER CSD2007-00015].

*Conflict of interest statement*. None declared.

## Supplementary Material

Supplementary Data
