# Supplementary material for: DNA-binding determinants promoting NHEJ by human Polµ
Source: Nucleic Acids Res. 2012 Oct 2;40(22):11389–403. doi: 10.1093/nar/gks896 (PMC3526283; doi:10.1093/nar/gks896)
Supplement: Supplementary Data [file supp_40_22_11389__index.html]

DNA-binding determinants promoting NHEJ by human Polµ — DNA-binding determinants promoting NHEJ by human Polµ — Supplementary Data 

# DNA-binding determinants promoting NHEJ by human Polµ

## Supplementary Data

files

**Files in this Data Supplement:**

- Supplementary Data - pdf file
