# Supplementary material for: DNA-binding determinants promoting NHEJ by human Polµ
Source: Nucleic Acids Res. 2012 Oct 2;40(22):11389–403. doi: 10.1093/nar/gks896 (PMC3526283; doi:10.1093/nar/gks896)
Supplement: Supplementary Data [file supp_gks896_nar-00917-d-2012-File011.pdf]

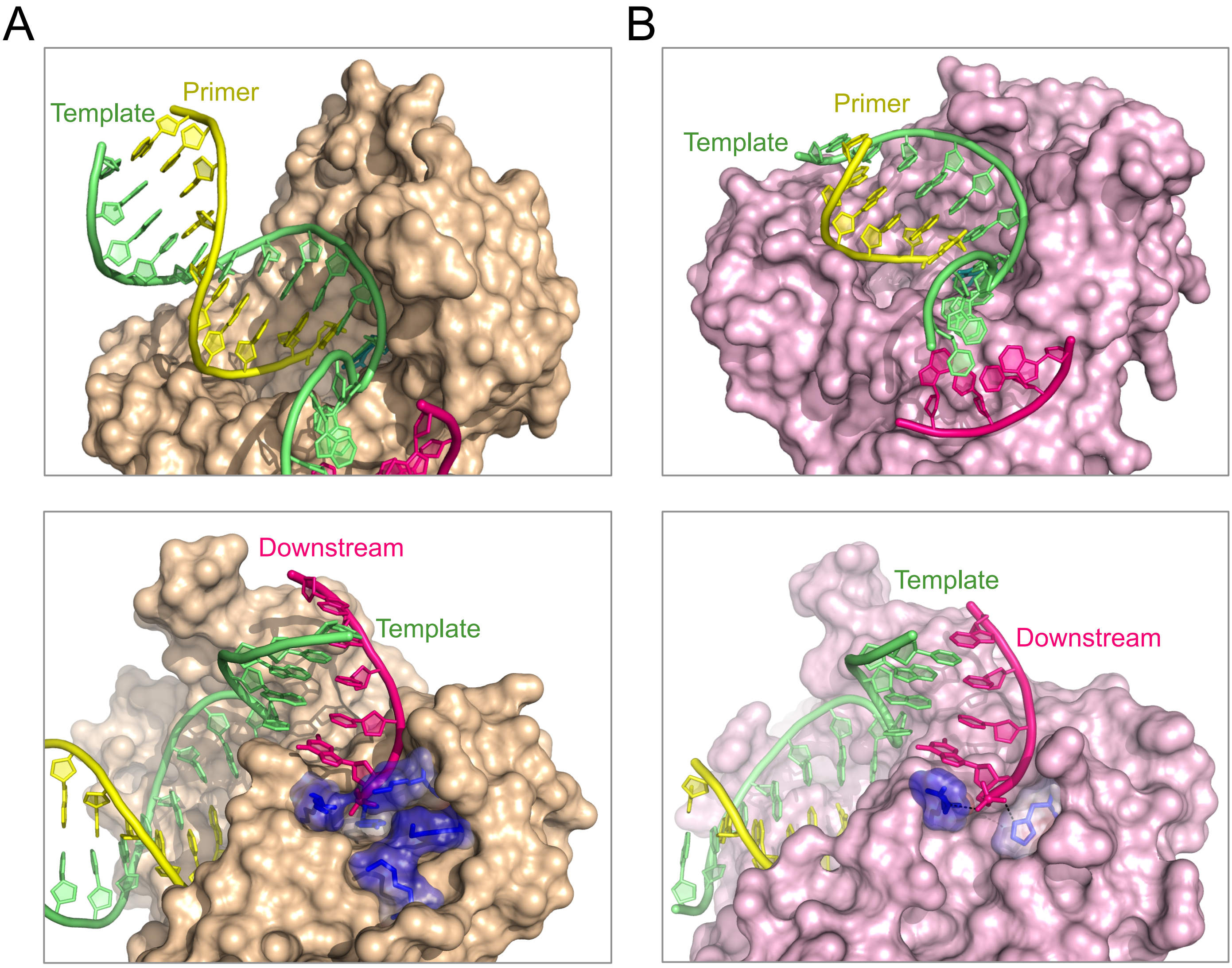

Supplementary Figure 1

Supplementary figure 1. A) Views of the crystal structure of Polß ternary complex (1BPZ) in which the protein is shown in wheat-colored surface, the DNA substrate is shown in sticks with the template strand in green, the primer strand in yellow and the downstream strand in dark pink. The incoming dNTP is shown in dark teal. B) Views of the crystal structure of Polµ ternary complex (2IHM) in which the protein is shown in pink-colored surface and the DNA and NTP substrates are shown as in A). In both cases, the residues forming the 5'P pocket are depicted in blue sticks with the electrostatic surface shown in a semi-transparent mode.

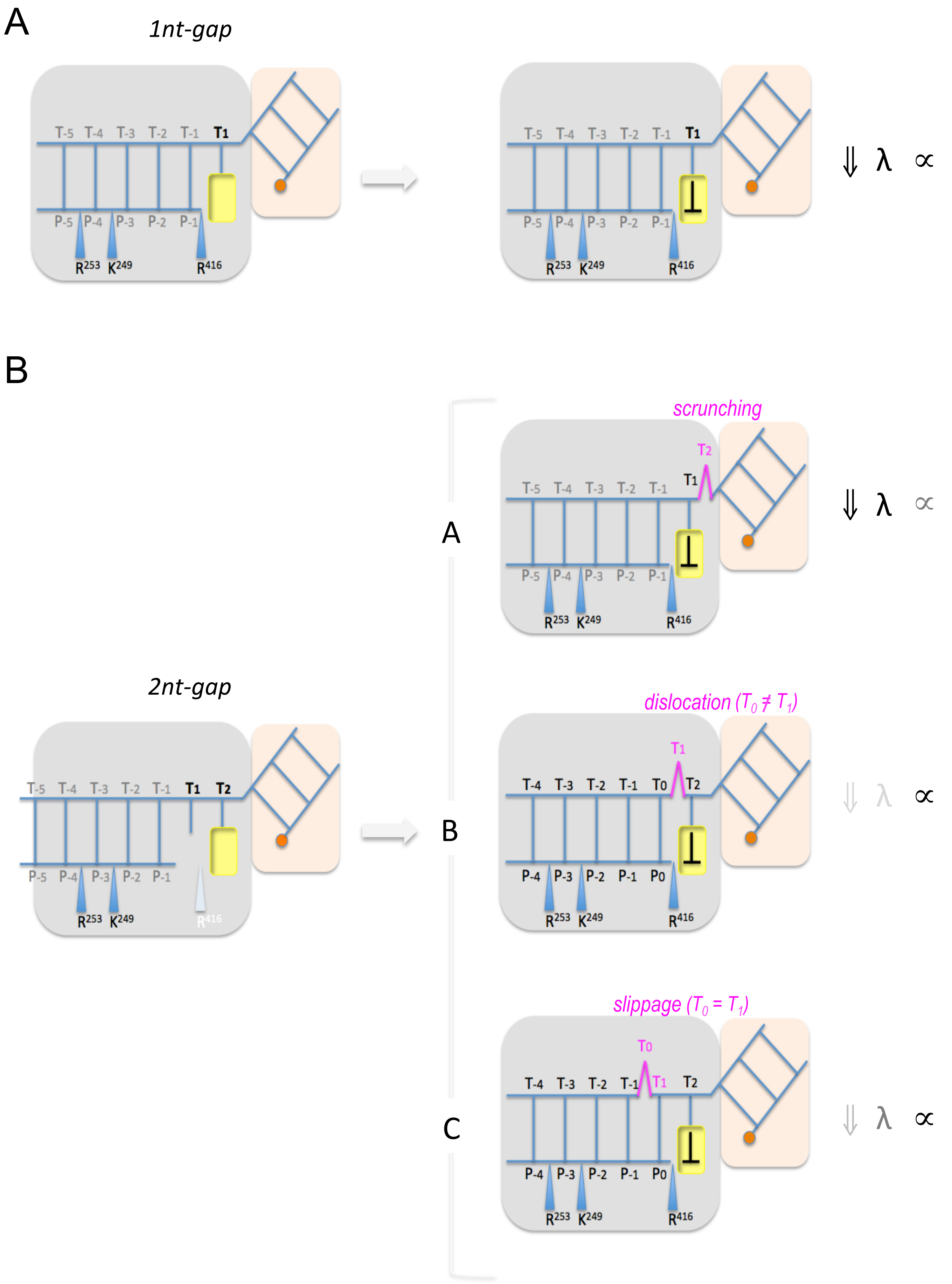

Supplementary Figure 2

Supplementary figure 2. A) Cartoon of the binary and ternary complexes of Polμ with a 1 nt gapped substrate and incoming nucleotide, showing the interactions of the residues Lys^{249}, Arg^{253} and Arg^{416} with the primer strand. The Polß-like core of the polymerase is shown as a grey rectangle while the 8 kDa domain is shown as an orange rectangle. The 5'P group of the downstream strand is shown as an orange circle. B) Cartoon representation of the predicted initial binding of Polμ to a 2 nt gap. Residues Lys^{249} and Arg^{253} are shown in blue, interacting with the primer strand, while residue Arg^{416} is shown in light blue/white, since it is not establishing any interactions with the 3'-primer terminus. The three possible mechanisms for polymerization on a gap longer than 1 nt are shown at the right. Scheme A illustrates the *scrunching* mechanism, with the formation of a bubble in the template strand (in this case containing only one flipped-out nucleotide (T_2) downstream to the polymerization site. This mechanism has been observed for Polλ in several crystal structures (31). Scheme B shows the *dislocation* mechanism, in which the bubble formed in the template strand is located immediately upstream to the polymerization site. The T_1 nucleotide, the first templating base, is flipped-out and T_2 is directing incorporation of the incoming nucleotide, and thus a frameshift is being generated. In scheme C the *slippage* mechanism is depicted. This last option also implies the formation of a bubble in the template strand upstream to the polymerization site, but in this case maintained by the new pairing stablished between the 3' terminus of the primer and the T_1 position, given that T_0 and T_1 bases in the template strand are identical.

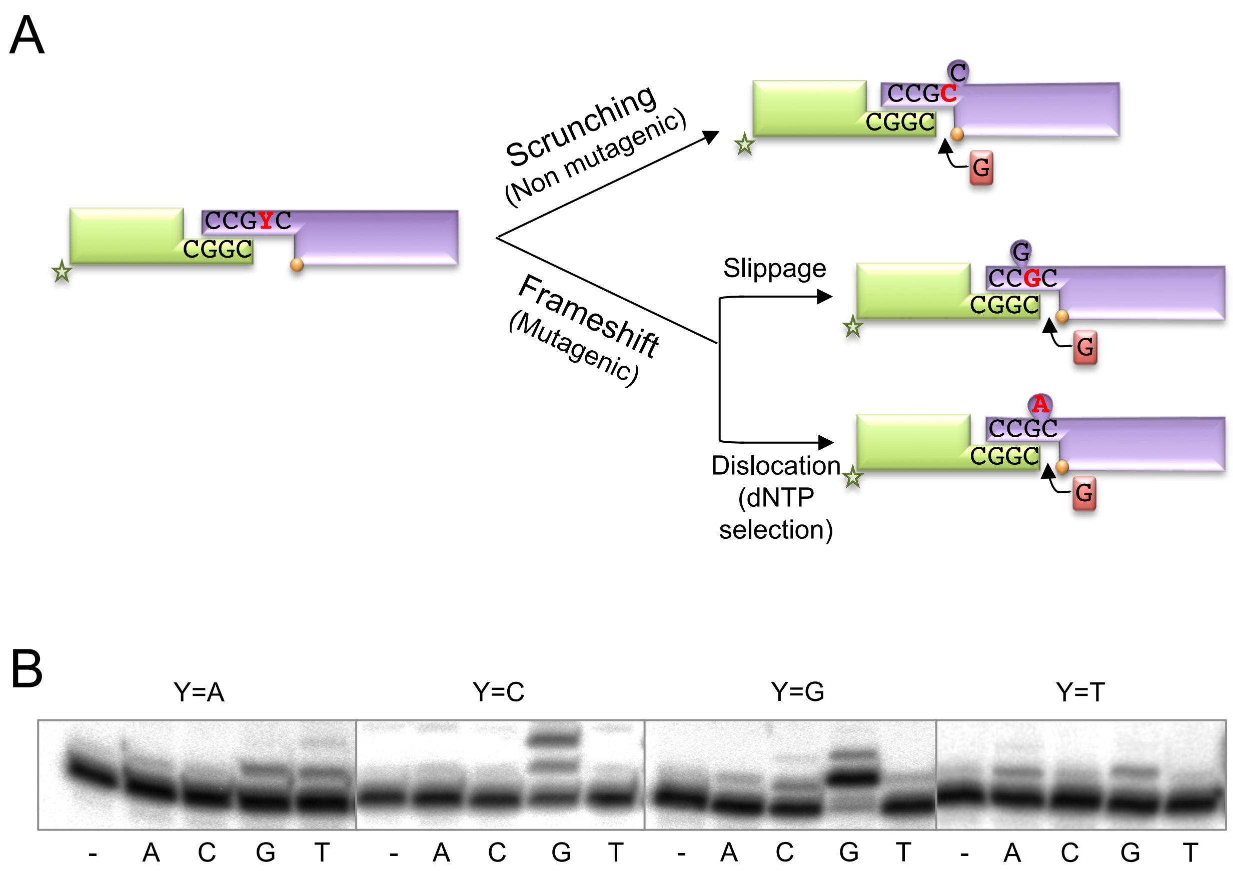

Supplementary Figure 3

Supplementary figure 3. Frameshifts during Polμ-mediated NHEJ. A) Schemes of the mechanisms by which Polμ can deal with a long gap during NHEJ. B) NHEJ reactions performed as described in Materials and Methods, with 200 nM Polμ and a set substrates shown in A), formed by hybridization of D3 with D1 (labeled substrate, shown in green) or by hybridization of either D4AC, D4CC, D4GC or D4TC with D2 (cold substrates, shown in purple). The orange spheres indicate the presence of a 5'P group in the downstream strand of the cold substrate. When indicated, dNTPs were added separately at 10 μM in the presence of 2.5 mM $MgCl_2$. After electrophoresis, the labeled fragments were detected by autoradiography.

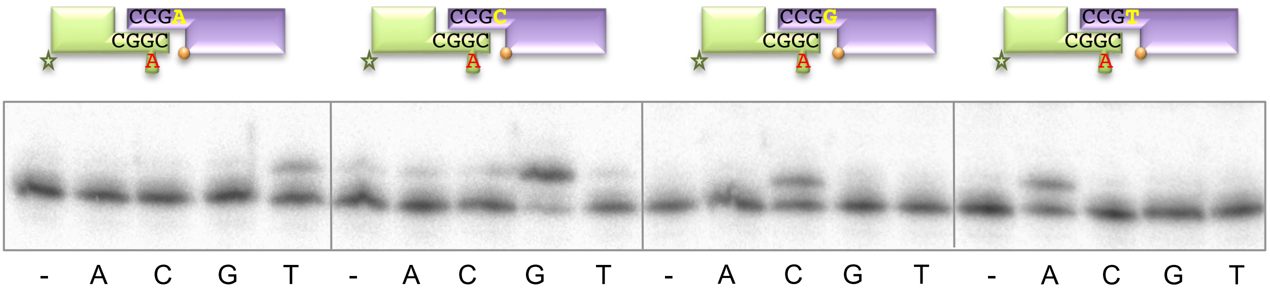

Supplementary Figure 4

Supplementary figure 4. Polµ-mediated NHEJ: non-aligned ends. NHEJ reactions performed as described in Materials and Methods, with 200 nM Polµ and using a set substrates formed by hybridization of D3BB1 with D1 (labeled substrate, shown in green) or by hybridization of either D4A, D4C, D4G or D4T with D2 (cold substrates, shown in purple). The orange spheres indicate the presence of a 5'P group in the downstream strand of the cold substrate. When indicated, dNTPs were added separately at 10 µM in the presence of 2.5 mM $MgCl_2$. After electrophoresis, the labeled fragments were detected by autoradiography.

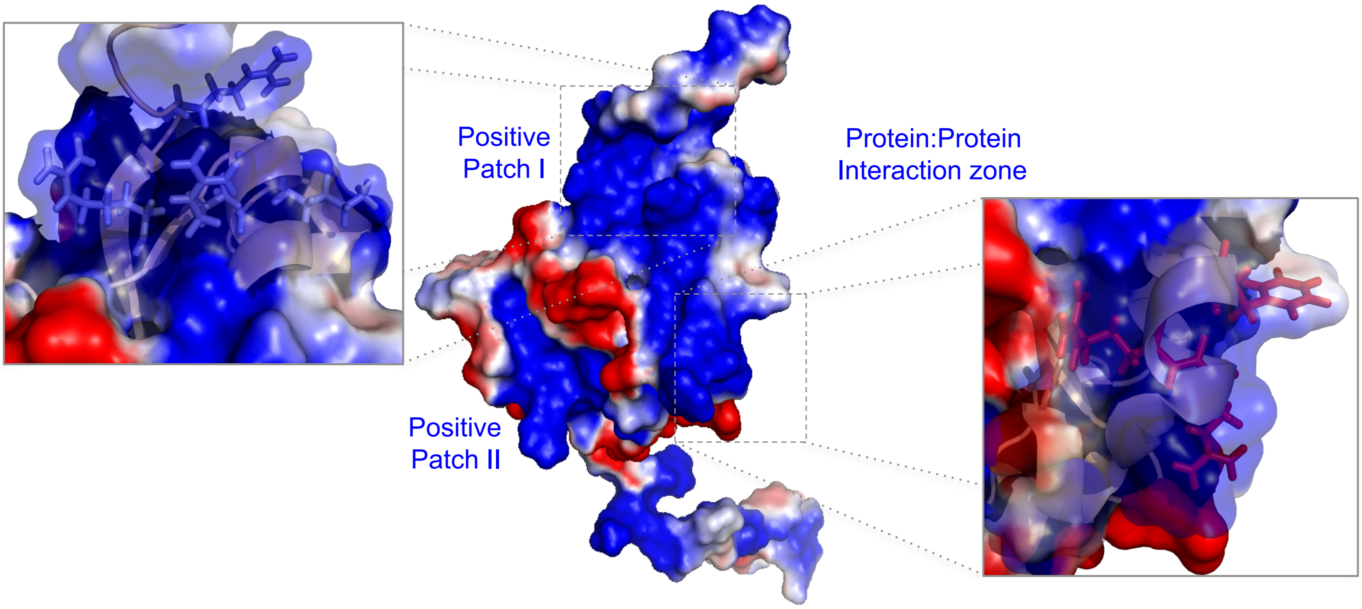

Supplementary Figure 5

Supplementary figure 5. BRCT domain of Polµ: surfaces of interaction. Electrostatic surface representation of the BRCT domain of Polµ (PDB ID: 2DUN). Two positive patches are indicated in the same face of the domain. The inlay to the right shows a close-up of the surface of interaction of the BRCT domain with other NHEJ factors, in the opposite face of the domain, while the inlay to the left shows a close-up of the predicted surface of interaction of the BRCT with the DNA substrate, that corresponds to positive patch I.

	Km_{app} (μM)	Kcat (s^{-1})	Catalytic efficiency
Gap 5'P	**86,23** ± 3,80	**0,0030** ± 0,00076	$\mathbf{3,5 \times 10^{-5}}$ ± $8,4 \times 10^{-6}$
Gap 5'OH	**382,92** ± 33,82	**0,0030** ± 0,00028	$\mathbf{7,9 \times 10^{-6}}$ ± $1,2 \times 10^{-6}$

Supplementary table 1. Comparison of the kinetic parameters of wild type Pol*μ* polymerization on a 1 nt gapped substrate either having or lacking a 5'P group at the downstream strand. The values for the Km_{app}, K_{cat} and catalytic efficiency are an average of three independent experiments.
